# Molecular and spatial analysis of tertiary lymphoid structures in Sjogren’s syndrome

**DOI:** 10.1038/s41467-024-54686-0

**Published:** 2025-01-02

**Authors:** Saba Nayar, Jason D. Turner, Saba Asam, Eanna Fennell, Matthew Pugh, Serena Colafrancesco, Onorina Berardicurti, Charlotte G. Smith, Joe Flint, Ana Teodosio, Valentina Iannizzotto, David H. Gardner, Joel van Roon, Ilya Korsunsky, Dawn Howdle, Roche Fibroblast Network Consortium, Michael Brenner, Mark Coles, Fiona M. Powrie, Soumya Raychaudhuri, Andreas P. Frei, Kara G. Lassen, Andreas P. Frei, Kara G. Lassen, Simon J. Bowman, Wan-Fai Ng, Adam P. Croft, Andrew Filer, Benjamin A. Fisher, Christopher D. Buckley, Francesca Barone

**Affiliations:** 1 Rheumatology Research Group, Department of Inflammation and Ageing, College of Medicine & Health, University of Birmingham, Birmingham, UK; 2 National Institute for Health Research (NIHR) Birmingham Biomedical Research Centre, University Hospitals Birmingham NHS Foundation Trust, Birmingham, UK; 3 Birmingham Tissue Analytics, College of Medicine & Health, University of Birmingham, Birmingham, UK; 4 UCL Genomics, Zayed Centre for Research into Rare Disease in Children, University College London, London, UK; 5 School of Medicine & HRI & Bernal Institute, University of Limerick, Limerick, Ireland; 6 Department of Immunology and Immunotherapy, College of Medicine & Health, University of Birmingham, Birmingham, UK; 7 Department of Biomedical Sciences, Humanitas University, Milan, Italy; 8 Rheumatology, Immunology and Clinical Medicine Unit, Department of Medicine, Università Campus Bio-Medico, Rome, and Immunorheumatology Unit, Fondazione Policlinico Universitario Campus Bio Medico, Rome, Italy; 9 Department of Rheumatology & Clinical Immunology/Laboratory of Translational Immunology, University Medical Centre Utrecht, Utrecht University, Utrecht, The Netherlands; 10 Center for Data Sciences, Brigham and Women’s Hospital, Boston, MA USA; 11 Roche Pharma Research and Early Development, Roche Innovation Center Basel, F. Hoffmann-La Roche Ltd, Basel, Switzerland; 12 HRB Clinical Research Facility, University College Cork, Cork, Ireland; 13 Kennedy Institute of Rheumatology, University of Oxford, Oxford, UK; 14 Candel Therapeutics, Needham, MA USA; 15 Division of Rheumatology, Inflammation and Immunity, Department of Medicine, Brigham and Women’s Hospital and Harvard Medical School, Boston, MA USA

**Keywords:** Imaging the immune system, Lymphoid tissues, Autoimmunity, Systems analysis

## Abstract

Tertiary lymphoid structures play important roles in autoimmune and non-autoimmune conditions. While many of the molecular mechanisms involved in tertiary lymphoid structure formation have been identified, the cellular sources and temporal and spatial relationship remain unknown. Here we use combine single-cell RNA-sequencing, spatial transcriptomics and proteomics of minor salivary glands of patients with Sjogren’s disease and Sicca Syndrome, with ex-vivo functional studies to construct a cellular and spatial map of key components involved in the formation and function of tertiary lymphoid structures. We confirm the presence of a fibroblast cell state and identify a pericyte/mural cell state with potential immunological functions. The identification of cellular properties associated with these structures and the molecular and functional interactions identified by this analysis may provide key therapeutic cues for tertiary lymphoid structures associated conditions in autoimmunity and cancer.

## Abstract (web summary)

Tertiary lymphoid structures play important roles during homeostatic but also immunopathological conditions including autoimmune disorders. Here the authors integrate single cell sequencing with spatial proteomics and transcriptomics to define a cellular and spatial map of tertiary lymphoid structures in salivary glands of patients with Sjogren’s syndrome.

## Introduction

Tertiary lymphoid structures (TLS) provide a functional microenvironment for activated T and B cells to reside, proliferate and differentiate within non-lymphoid tissues^1–9^. Whilst unlikely to play a role in disease initiation, TLS support disease persistence and have been shown to associate with clinical features, response to therapy and progression in a range of autoimmune diseases and in cancer^10^. Associations with pathophysiology have been most clearly demonstrated in Sjögren’s syndrome (SjS), a disease where formation of TLS and maturation of ectopic germinal centers in lymphoid structures correlate with B cell hyperactivity, autoimmunity, pathogenic humoral response and development of B cell lymphoma^5,11–17^.

We and others have previously demonstrated that FAP^+^PDPN^+^ fibroblasts, a population of tissue-resident fibroblasts that share features and functions with fibroblastic reticular cells inhabiting secondary lymphoid organs (SLOs), define the anatomical framework for TLS establishment and maintenance in peripheral tissue^5,13,18,19^. Analysis of murine models of TLS and human samples from patients with SjS has unveiled multiple signals and molecular mechanisms involved in the activation of this population of fibroblast named immunofibroblasts, leading us to propose the presence of a PDGF-Rα^+^/PDGF-Rβ^+^ tissue-resident progenitor cell that differentiates in response to local cues, to acquire novel immunological properties supporting TLS establishment^5^. Inflammatory and lymphoid cytokines such as IL-13, IL-22 and both lymphotoxin alpha and beta (LTα3 and LTα1β2) have been shown to play a role in the activation and differentiation of immunofibroblasts^5^.

Use of computational biology approaches applied to single-cell transcriptomic data has provided novel cellular atlases of healthy and disease tissues and molecular signatures to identify gene cassettes, enabling the categorization of fibroblast clusters that share similar features across different diseases and in different organs^20,21^. Using cellular interaction inference tools within scRNAseq data, investigators have also been able to propose molecular interactions between specific cell populations. An example of these findings is the identification of the role of NOTCH3 in establishing a transcriptional gradient for fibroblast differentiation in the synovial tissue in Rheumatoid Arthritis^22^ and the discovery of the complement component C3 as a mediator in macrophage/cancer-associated fibroblast crosstalk^23^. The complexity of the stromal cell compartment of murine lymph nodes (LN) has been also deconvoluted, unveiling an unexpected level of heterogeneity and cellular specialization within lymph nodes^24^. Different populations of fibroblasts create specialized micro domains within the LN, which influence antigen presentation, cell migration, retention, activation and survival of T and B lymphocytes^11,25–30^, all of which ultimately shape the immune response.

So far, human tissues fostering TLS structures have not been thoroughly explored at single-cell resolution, leaving an important gap in our understanding of the cellular and molecular properties involved in TLS establishment, maintenance, and evolution. Importantly, as for other complex dynamic microenvironments, TLS single cell analysis needs to be integrated within the context of TLS histological organization to provide a developmental reference for disease pathophysiology. Here we have determined the cellular and spatial basis of TLS formation in SjS to unveil TLS-specific features, identifying cellular players, delineating key pathways involved in cellular cross talk and defining the developmental trajectory of TLS formation and function in peripheral tissue. This study, whilst providing the first comprehensive map of TLS cellular and molecular properties, provides tools to promote understanding of these structures in disease and to select specific modulators for TLS manipulation in human disease.

## Results

### Single cell analysis unveils the complex cellular landscape of TLS in human salivary gland

**Fig. 1:**
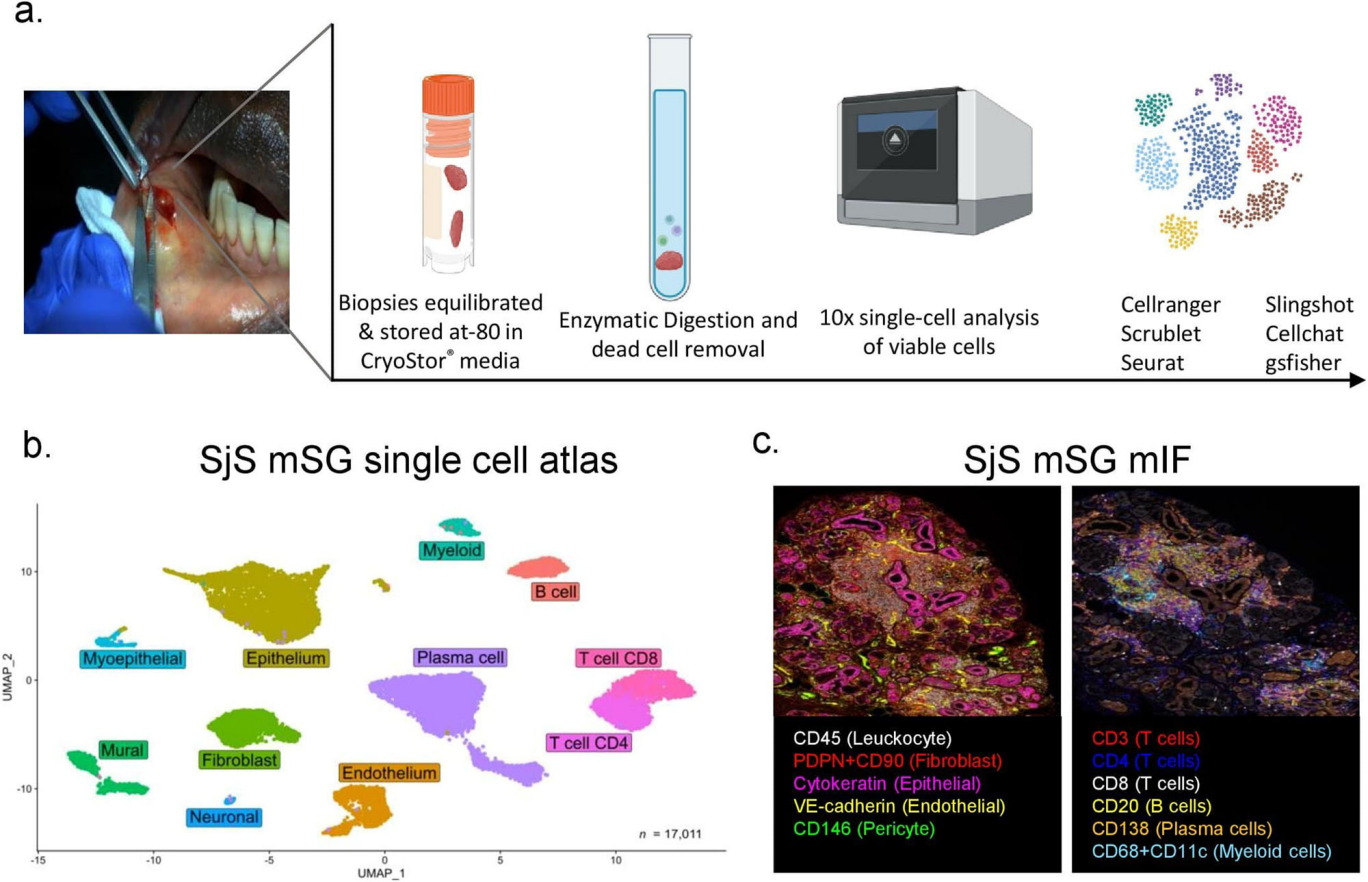
Cellular landscape of the minor salivary glands of SjS patients. **a** Minor salivary gland (mSG) biopsy 10x single cell workflow pipeline, Created in BioRender. *NAYAR, S. (2024)* https://BioRender.com/l06g537. **b** Uniform manifold approximation and projection (UMAP) embedding of 17,011 single cells and gross-cell identities clustering of single-cell gene expression data from *n* = 7 Sjogren’s syndrome (SjS) salivary gland samples illustrating both immune and stromal cell clusters. **c** Multiplex immunofluorescence image illustrating major lineages identified in minor salivary gland tissue from Sjogren’s patients (*n* = 3) probed with a 6-plex panels for CD146 (green), VE-cadherin or CD20 (yellow), Pan-Cytokeratin/CK (magenta), CD138 (orange), and CD68 + CD11c (cyan), CD45 or CD8 (white), Podoplanin/PDPN+CD90 or CD3 (red) and CD4 (blue), Scale bar = 50 µm.

To gain single-cell resolution of the inflamed glandular microenvironment in salivary glands (SG) harboring TLS and to better understand cellular properties associated with ectopic lymphoneogenesis, we performed 10x Single-cell RNA sequencing on labial minor SG biopsies obtained from seven patients with SjS (supplementary Data 1; 17,011 cells passed QC) (Fig. 1a). Within the global cellular landscape of the human SjS SG, we identified 11 main cell clusters consisting of 5 hematopoietic and six stromal cell clusters. In the *CD45*^–^ stromal cell compartment we identified epithelial/myoepithelial, endothelial, fibroblast, mural, and a small neuronal cell population. Subclustering of the *CD45*^+^ hematopoietic compartment unveiled presence of plasma cells, B cells, CD4^+^ and CD8^+^ CD3^+^T lymphocytes, and myeloid cells (Fig. 1b). Multiplex immunofluorescence with differentiating markers for each of the identified cell types (Fig. 1c and supplementary Fig. 1), combined with analysis of gene cassettes associated with each cell type (supplementary Fig. 1, supplementary Data 2), confirmed our annotation of the hematopoietic and stromal populations. Cluster distribution was validated across all analyzed samples (supplementary Fig. 1c).

### Stromal cell clustering reveals significant fibroblast diversity and an immunomodulatory pericyte/mural cell population in Sjögren’s syndrome

**Fig. 2:**
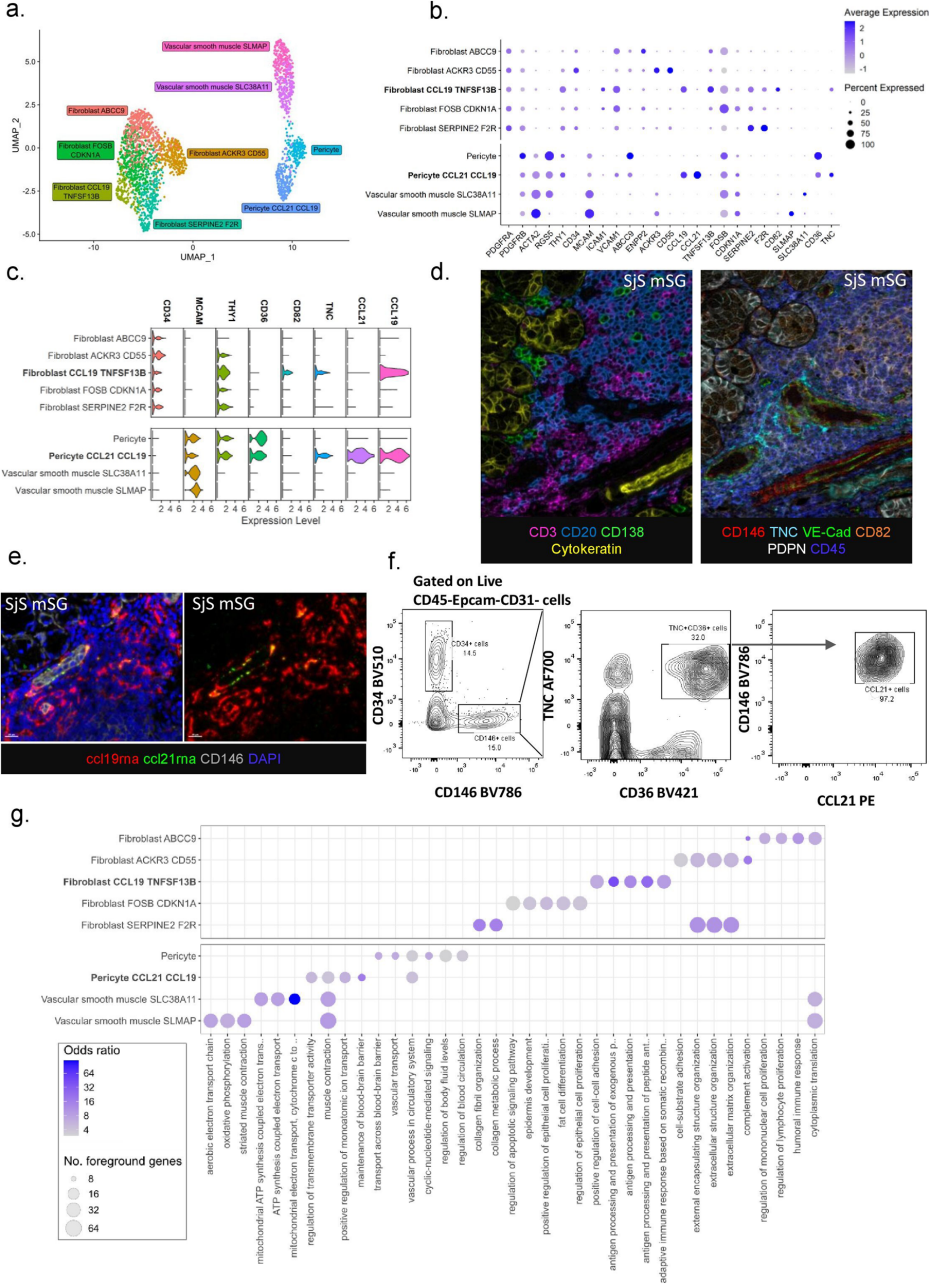
Single cell resolution of fibroblast and mural cell subsets in human SjS salivary glands. **a** Uniform manifold approximation and projection (UMAP) dimensional reduction and sub-clustering of fibroblast and mural cell transcriptomic data in Sjogren’s syndrome (SjS) minor salivary glands (mSG). **b** Expression of current identifying and newly discovered identifying genes across the fibroblast and mural subsets. **c** Expression of gene cassettes used to spatially identify immunofibroblast and CCL21 CCL19 pericytes in salivary glands. **d** Multiplex immunofluorescence image illustrating immune cells and pericytes identified in minor salivary gland tissue (*n* = 2) probed with a 6-plex panels for CD138 (green), CD20 or TNC (yellow), Pan-Cytokeratin/CK or CD82 (orange), VE-cadherin (cyan), Podoplanin/PDPN (white), CD3 or CD146 (red) and DAPI or CD45 (blue), Scale bar = 20 µm. **e** Multiplex immunofluorescence image of CD146 (grey) pericytes with ccl21rna (green), ccl19rna (red) and DAPI (blue) in immune aggregate within human salivary glands of patients (*n* = 2) with Sjögren’s syndrome, Scale bar = 20 µm. **f** Representative flow cytometric identification of CD146+TNC+ CD36+CCL21+ pericyte cell population in Sjogren’s syndrome minor salivary glands. **g** Gene-set overrepresentation analysis (using GO and Kegg gene sets) of the fibroblast and mural populations, the top 5 enriched gene sets for each cell subset are shown.

We and others have previously demonstrated that fibroblasts are key in supporting TLS development and maintenance in the tissue^5,10,13,23,31–35^. However, heterogeneity is present in this compartment, that must be resolved, to develop the ability to manipulate TLS establishment. To this end, we performed sub-clustering on *CD45*^-^ cell clusters identifying multiple clusters within the fibroblast and mural compartments amongst others (Fig. 2a). Extensive differential expression analysis was implemented, to validate the cellular identity of the annotated clusters (supplementary Fig. 2a, supplementary Data 3) and key differentiating genes, identified using the implementation of the MAST algorithm within Seurat, as summarized in Fig. 2b.

Fibroblasts with immunological functions have been previously described both in humans and mice as CD45^–^ cells expressing ICAM-1, VCAM-1, podoplanin (PDPN) and CD34^5^. Within the fibroblast clusters, we identified the presence of a population defined by the expression of *CD34*, *CCL19*, *TNFSF13B*, *ICAM1*, *VCAM1*, *CD82*, and *CXCL9* displaying key overlapping features consistent with the phenotype of previously described immunofibroblasts^5^. Next, we identified a cluster defined by the expression of *CD55*, *C1R* and *ACKR3*, a chemokine scavenger receptor likely involved in the establishment of chemokine gradients within the TLS complex microdomains^36^. The potential role of this cluster in the cross talk with neighboring cell types was supported by the expression of the complement receptor *CD55* and *C1R*. Within the remaining three fibroblast subclusters, we identified a *SERPINE2* expressing subcluster, displaying expression of *CLEC14A*, *F2R* and elevated transcripts for *DKK3* indicating a potential engagement of Wnt signaling pathways. We also identified a *FOSB CDKN1A* expressing subcluster, displaying evidence of activation of the AP-1 pathway with increased *FOSB*, *FOS*, *JUND*, and *JUN* expression. Finally, an *ABCC9* fibroblast subcluster was recognized, defined by the expression of *APOC1*, *APOE*, *PLA2G2A*, and *CFD*, genes involved in lipid metabolism and alternative complement activation^37^ (Fig. 2b, supplementary Fig. 2a, supplementary data 3). We used slingshot trajectory analysis with UMAP reduction to order cells and infer relationships between these clusters (supplementary Fig. 2b). The *CD34*, *CCL19*, *TNFSF13B*, *ICAM1*, *VCAM1*, *CD82*, *CXCL9* (immunofibroblast or CCL19 TNFSF13B cluster) cluster showed the closest relationship to the fibroblast SERPINE2 F2R and fibroblast FOSB CDKN1A clusters. The fibroblast ACKR3 cluster occupied one end of the trajectory and displayed similarities to the PI16 universal reservoir fibroblast population identified by ref. ^21^ including expression of *PI16*, *ACKR3*, *ACKR1*, *MFAP5*, *PCOLCE2*, *CD248*, and *IGFBP6*. Furthermore, the ACKR3 cluster displayed signatures associated with stemness, suggesting that the ACKR3 cluster might act as precursor of all the other fibroblast clusters. The ABCC9 and FOSB populations are the least distinct in terms of marker profile. They may represent activation or intermediate states between the other three populations, suggesting their nature as transitional/intermediate populations.

An additional stromal cell compartment was identified, characterized by the expression of *ACTA2* and therefore associated with mural cells or pericytes. Within this compartment, we classified 4 clusters of cells which comprised of 2 pericyte clusters and 2 vascular smooth muscle cell clusters (Fig. 2a–c). Both pericyte clusters expressed the classical markers *RGS5*, *ACTA2*, and *MCAM*; however, one cluster was further characterized by the expression of the lymphoid chemokines *CCL21* and *CCL19* and the glycoprotein tenascin C (*TNC*) (Fig. 2b, c). In contrast to other pericyte clusters, this CCL21^++^ CCL19^++^ TNC^+^ cluster expressed *CCL8*, *VCAM1*, and elevated *CCL2* gene transcripts (Fig. 2b and supplementary Fig. 2a). Whilst the CCL21^++^ CCL19^++^ TNC^+^ pericyte cluster expressed key chemokines implicated in TLS formation, it lacked expression of *TNFSF13B*, *CD82* and *PDGFRA* classical of the CCL19 TNFSF13B fibroblast.

We extrapolated gene expression cassettes from single-cell profiling and used a combination of markers to identify and validate the presence of the fibroblast and pericyte subclusters in tissue using multiplex immunofluorescence (Fig. 2c,  d), RNA scope, and flow cytometry on tissue samples and digested SjS SG biopsies (Fig. 2e, f and supplementary Fig. 2). Histological analysis demonstrated enrichment of CCL21+ pericytes/mural cells in proximity to peripheral node addressing (PNAd^+^) high endothelial venules, confirming the peri-vascular nature of these cells and suggesting a potential immunological role for this population in recalling T cells within the TLS for this population (supplementary Fig. 2e). To evaluate the developmental relationship between these fibroblasts and pericytes/mural cells with immunological function, we applied a fibroblast progenitor module score across fibroblast and mural populations, using the set of genes identified by ref. ^21^. CCL21 CCL19 pericytes were found not to score highly in this fibroblast progenitor module, suggesting that they are unlikely to represent a stem fibroblast population in SG TLS (supplementary Fig. 2c).

Finally, to further dissect the distinct role of these different populations in TLS, we performed gene-set overrepresentation analysis to identify differentially expressed pathways in pericytes/mural cells and fibroblasts presenting immunological functions. The top five enriched gene sets for each cluster are represented in Fig. 2g. The CCL19 TNFSF13B cluster showed enrichment for genes regulating antigen presentation and response to interferon gamma, reflecting the proinflammatory nature of this population. The fibroblast SERPINE2 F2R cluster was enriched for genes responsible for response to growth factors, ossification, and collagen metabolism. Fibroblast ABCC9 displayed enrichment of pathways involved in protein targeting endoplasmic reticulum. Fibroblast FOS CDKN1A showed enrichment for response to steroids and apoptosis. Finally, the ACKR3 CD55 cluster showed enrichment in the regulation of angiogenesis and enrichment for complement activation pathways. Conventional pericytes display enrichment for pathways responsible for sodium excretion; however, the pericyte population expressing *CCL21* and *CCL19* showed enrichment in pathways including transmembrane transport and ion transport, but not limited to sodium excretion.

### Different signals underpin the production of CCL21 and CCL19 in fibroblasts compared to pericytes/mural cells

**Fig. 3:**
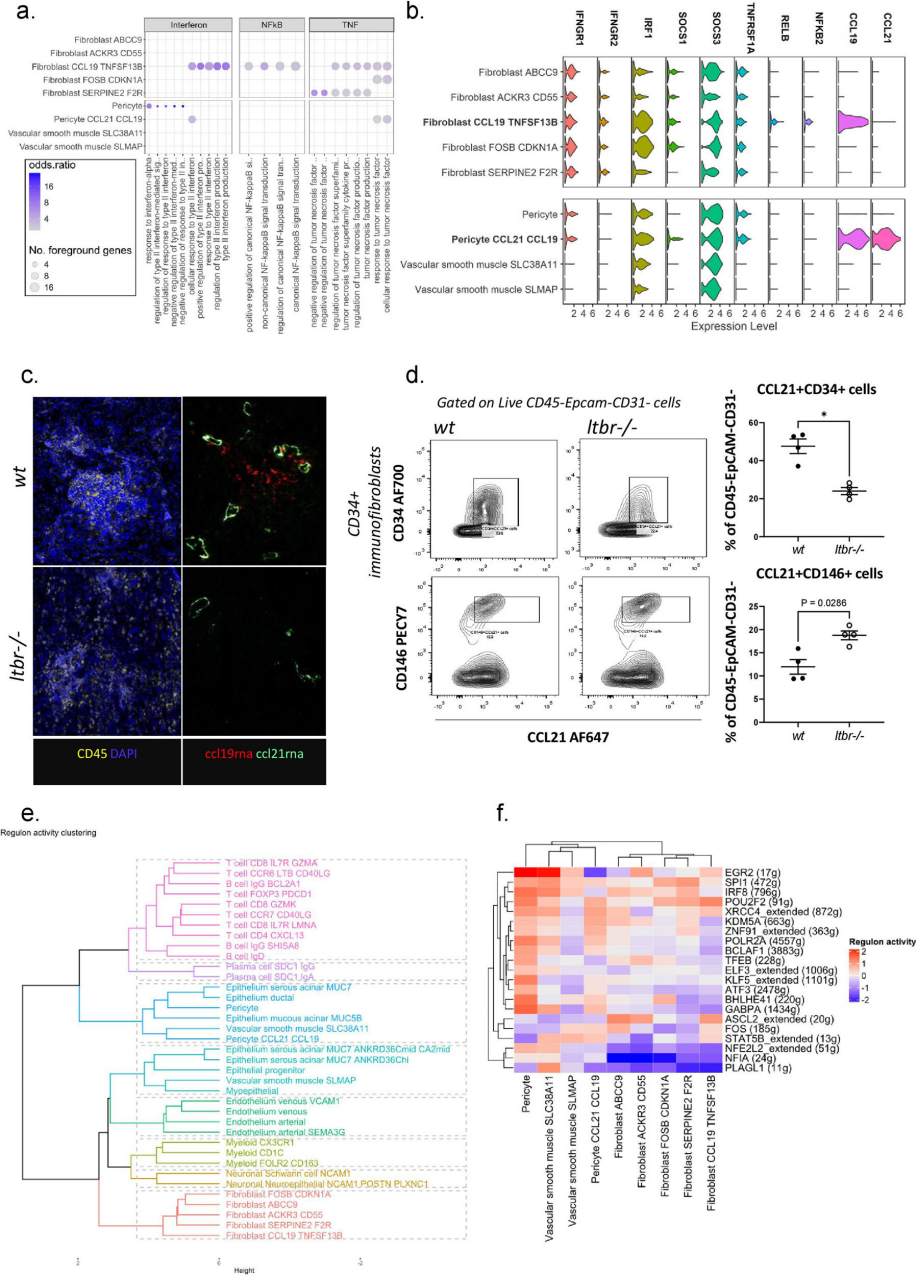
Immunofibroblasts and CCL21 CCL19 pericytes show differential dependence on signalling pathways. **a** Select pathways from the gene-set overrepresentation analysis (using GO and Kegg gene sets) of fibroblast and mural populations highlighting the enrichment of interferon and TNF pathways in both immunofibroblasts and CCL21 CCL19 pericytes, and NFκB pathway enrichment restricted to immunofibroblasts. **b** Violin plot of expression of genes related to the enriched pathways shown in Fig. 3a across fibroblast and mural clusters. **c** Multiplex immunofluorescence image of ccl21rna (green), ccl19rna (red) vascular expression in DAPI (blue) and CD45^+^ (yellow) immune aggregate in TLS-induced salivary glands of wildtype (*wt*) and lymphotoxin beta receptor knockout (*Ltbr*^*–/–*^) mice, *n* = 2 per group, Scale bar = 100 µm. *Ltbr*^*–/–*^ results in abrogation of TLS and decreased CCL19 expression within immune aggregates. Perivascular expression of CCL19 remained in the *Ltβr*^*–/–*^*.* **d** Flow cytometric analysis of TLS-induced salivary glands of wildtype (*wt*) and *Ltbr*^*–/–*^ mice for CCL21+CD34+ immunofibroblast and CCL21^+^CD146^+^ pericytes to confirm the dependence of immunofibroblasts on LTβR signalling and CCL21 CCL19 pericyte independence. Data are mean ± s.e.m from two independent experiments with two mice analyzed per group. Number of asterisks indicate significance: *p < 0.05, Mann–Whitney test, two-tailed. **e** Clustering of cell type by SCENIC derived regulon activity displaying distinct clustering of the fibroblast and mural lineages. **f** Heatmap of the regulon activity of the top 15 regulons by activity for the fibroblast CCL19 TNFSF13B and the pericyte CCL21 CCL19 clusters.

Intriguingly, whilst sharing the ability to secrete T-cell chemokines, pericyte/mural cell gene-set profiling was significantly different from that of fibroblasts expressing these chemokines. Importantly, NFkB gene-set enrichment was restricted to the CCL19 TNFSF13B clusters subcluster (Fig. 3a). Accordingly, expression of *RELB* and *NFKB2* was limited to the CCL19 TNFSF13B clusters, confirming that only this population of fibroblasts, but not that of the CCL21 CCL19 pericyte/mural cell, rely on the lymphotoxin β receptor (LTβR) signaling pathway to produce lymphoid chemokines (Fig. 3b). The CCL21 CCL19 pericyte/mural cells, on the contrary, show no enrichment in the genes of the non-canonical NFKB signaling pathway, and expressed both TNFα and IFNγ associated genes, such as *IFNGR1*, *IFNGR2*, *TNFRSF1A*, *IRF1* and *SOCS1* (Fig. 3b). We have previously demonstrated that in *ltβr*^*–/–*^ mice, the production of the lymphoid chemokines CCL19 and CCL21 was abrogated immunologically active fibroblasts^5^. However, at the time of our studies, we noted that *Ccl21* expression was unexpectedly maintained in vascular/perivascular structures, suggesting that LTβR signaling is not required to support perivascular expression of this T cell chemokine^5,38,39^ (Fig. 3c and supplementary Fig. 3). We have now explained this observation, by quantifying the presence of the CCL21 CCL19 pericyte/mural cells and chemokine expressing fibroblasts in *wild type (wt) and ltβ*^*r–/–*^ murine SG infected with adenovirus to induce TLS^40^ (Fig. 3d). As expected from our previous findings, in the absence of LTβR (and engagement of the non-canonical NFKB pathway), the number of mature fibroblasts that expressed lymphoid chemokines significantly decreased (Fig. 3d). However, the number of pericytes/mural cells expressing CCL21 was not affected by LTβR abrogation, suggesting that, in mice as well as in our human dataset, the ability of this population to secrete lymphoid chemokines is independent from LTβR engagement. Due to the shared mesenchymal origin of pericytes and fibroblasts and the reported conversion between the two cell types we utilized SCENIC to infer coordinated gene regulon activity providing insight into potential transcription factor activity and the distinct identities of the two cell types. As shown in Fig. 3e hierarchical clustering based on calculated regulon activity finds all fibroblast subsets forming a discrete branch of the clustering. Both of our reported pericyte subsets and one of the vascular smooth muscle cell subsets form a discrete cluster with epithelial subsets. Moreover, the branch containing the pericyte clusters bifurcates from the fibroblast-containing branch at the initial split in hierarchical clustering. In Fig. 3f, a heatmap of top 15 regulons by activity for the pericyte CCL21 and fibroblast TNFSF13B clusters recapitulates the mural versus fibroblast bifurcation.

### Single cell analysis defines cellular and functional properties for SjS and Sicca syndrome

**Fig. 4:**
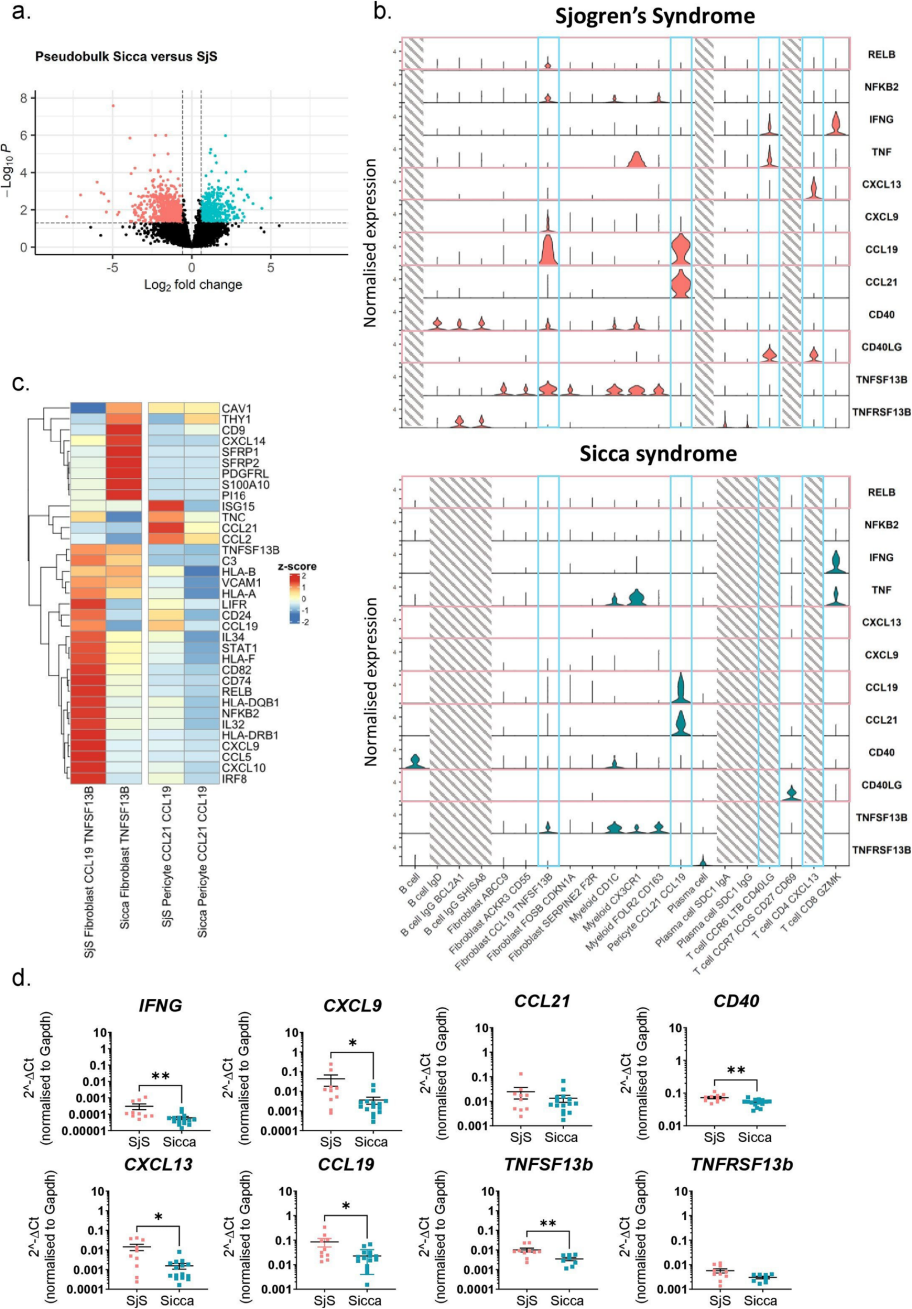
Signalling pathways differentiate between key populations in Sjogren’s syndrome and Sicca syndrome. **a** Volcano plot illustrating differentially expressed genes detected between pseudobulked Sjogren’s syndrome (SjS) and Sicca samples. Statistical testing completed using DESeq2 to fit a negative binomial generalised linear model with Wald test to calculate p-values and Benjamini-Hochberg method to correct for multiple comparisons. **b** Expression of genes related to signalling pathways and tertiary lymphoid structure formation in subsets from Sjogren’s syndrome or Sicca syndrome. **c** Select differentially expressed genes detected between single-cell clusters of immunofibroblasts or CCL21 CCL19 pericytes between SjS and sicca, data are plotted as z-scores of the summed aggregate of all samples for each cluster including all samples. **d** qPCR analysis for mRNA transcripts of *IFNG, CXCL9, CCL21, CD40, CXL13, CCL19, TNFSF13B and TNFSF13B* in in whole tissue RNA extracts from SjS and Sicca biopsies. mRNA transcripts were normalized to the housekeeping gene *GAPDH* RNA transcripts. Number of asterisks indicate significance: *p < 0.05; **p < 0.01, Mann–Whitney test, two-tailed. Data are representative as mean ± s.e.m, *n* = 10-15 (*IFNG, CXCL9, CCL21, CD40, CXL13 and CCL19)* and *n* = 8–10 *(TNFSF13B and TNFSF13B)*.

SjS patients share a series of symptoms with patients affected by Sicca syndrome, a SG disease characterized by dryness in absence of the hallmark antibody profile and aberrant B cell activation typical of SjS. Importantly, whilst often presenting a diffuse T cell infiltrate in the SG, patients with Sicca syndrome do not form TLS^41^. We used single cell analysis applied to 6 Sicca syndrome SG and compared their profile to the SjS dataset (*n* = 7) to dissect key cellular and functional properties of the two diseases. Pseudo-bulk analysis of the single cell data identified 1187 genes upregulated in SjS and 1016 genes upregulated in Sicca (Fig. 4a). Genes upregulated in SjS included *CXCL13*, *CXCR5*, *CD22*, *CD19*, *IGHG1*, *IL21, IFNG* and *SMR3B*. Genes upregulated in Sicca included *SCGB1D2*, *CAMP*, *SCGB2A1*, *S100A2*, and *LGALS7B*. In SjS, but not in Sicca samples, we identified a distinct *CCR6*^+^*LTB*^+^*BAFF*^+^ T helper cell cluster, a *CXCL13*^+^*CD40LG*^+^ T cell cluster and several B cell clusters (Fig. 4b and supplementary Fig. 4a, b). Notably, Limited expression of *CD40* and *CD40L* was observed on all cell types in Sicca; *CD40*, on the contrary, was broadly expressed on SjS cells, underlining the relevance this pathway in SjS pathology^42,43^.

Importantly, whilst CCL21 CCL19 pericytes/mural cells were found in Sicca (though with lower expression of *CCL19*), the CCL19 TNFSF13B fibroblast cluster was not detected in this condition. A population of fibroblasts expressing *TNFSF13B* but negative for the expression of the T cell attracting chemokines and *CD40* was present (Fig. 4b). Differential expression analysis among the two diseases (Fig. 4c) confirmed the diminished expression of the more complex CCL19 TNFSF13B fibroblast cluster signature in Sicca SGs, increased expression of WNT-signaling related genes and overall, a different engagement of key genes defining the function of these cells. Furthermore, comparative analysis of minor salivary gland fibroblast populations in disease (SjS and Sicca) and health (public dataset) found conserved clusters but with discrete shifts in subset maturation as expected (supplementary Fig. 4c). Bulk mRNA analysis from whole tissue extracted from SG of patients affected by either SjS or Sicca confirmed that *IFNG*, *CXCL9*, *CXCL13*, *CCL19*, *CD40*, and *TNFSF13B* were all expressed at significantly higher levels in SjS compared to Sicca (Fig. 4d). We performed functional experiments demonstrating that fibroblasts treated with IFNg and TNFa, two inflammatory mediators identified by our pseudobulk analysis, upregulated both CD40 and VCAM-1, showing that cues derived from the immune cells in the TLS microenvironment play a key role in influencing fibroblast functional phenotype (supplementary Fig. 4d). To expand this analysis and interrogate the reciprocal functional role of fibroblasts on the immune cells, we performed co-culture analysis that demonstrates how CCL19 TNFSF13B TLS fibroblasts are able to polarize T cell phenotype towards the Th1 lineage that release effector cytokines (IFNg and IL21), associated with TLS formation. Furthermore, we demonstrated that in presence of activated fibroblasts, the immune regulatory effect of cyclosporine, a commonly used immune suppressant, is abrogated, further supporting the functional role of this stromal cell population in supporting a pro-inflammatory microenvironment (Supplementary Fig 4e, f).

In addition, key differences in receptor/ligand interaction for both CXC and CC chemokines between in SjS and Sicca were identified by in silico analysis and summarized in supplementary Fig. 5a. In SjS we observed cross talk between the TNFSF13B clusters and haematopoietic cells that included TNFRSF13C/TNFSF13B (B cells), HLA-A,C and B with CD8A/B (T cells) and a range of collagens with CD44 (supplementary Fig. 5e). Interaction between fibroblast/pericyte derived MDK and T/B cell expressed NCL was also observed. The cross talk between TNFSF13B cluster and CCL21 pericytes/mural cells was mediated by CD44/collagen, ITGA1_ITGB1 (forming VLA-1) and NCL. Diversely, CCL21 pericytes/mural cells /hematopoietic cell cross talk was mainly mediated in SjS by HLA interaction with CD8. In Sicca we observed enriched interactions between the known inflammatory chemokine/chemokine receptor pair CXCL12/CXCR4, FGF7, APP and LAMA2 with CD44 and CD74 (T cells). CD44, ITGA1_ITGB1 and CD99 also mediated pericyte/fibroblast interaction in Sicca, while collagen/CD44 were mainly mediating interaction between pericytes and T cells in Sicca. All together these data demonstrate that disease specific molecular properties and cellular interactions in the two conditions reflect diverse stromal and hematopoietic cluster phenotypes in the two diseases, establishing distinct pathogenetic signatures in SjS and Sicca.

### Distinct molecular properties identify GC harboring in secondary and tertiary lymphoid structures

**Fig. 5:**
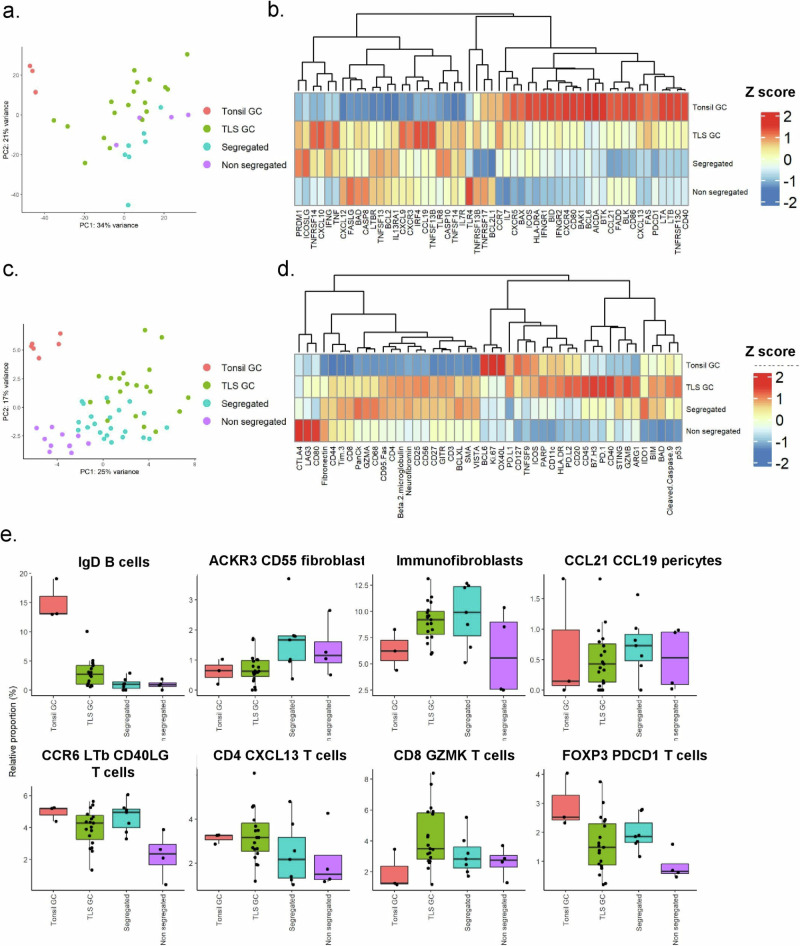
Bulk RNA-sequencing of microdissected regions of minor salivary glands from Sjogren’s syndrome patients and control tonsil tissue. **a** PCA plot of bulk-sequencing of microdissection samples. **b** Z-score mean expression of genes important to tertiary lymphoid organogenesis, in bulk-sequencing of microdissection data. **c** PCA plot of Nanostring Geomx protein expression data. **d** Z-score mean expression of proteins important to tertiary lymphoid organogenesis, in Nanostring Geomx data. **e** Cibersortx inferred cell subset proportions in the bulk sequencing samples using the single cell sequencing data as a reference atlas. Tonsil germinal center (GC) = 3, tertiary lymphoid structure germinal center (TLS GC) = 19, Segregated = 7, Non segregated = 4. Boxplots show the median, first and third quartiles, and whiskers extend to the largest value no further than 1.5 times the interquartile range.

TLS formation is a dynamic process. Small T cell aggregations occur first and are followed by progressive B cell infiltration and formation of germinal centers only occurs in mature, large TLS. It is known that lymphoid aggregates, commonly known in SjS as foci, at different stages of cellular organization are often found in the same SG tissue, limiting the ability of single-cell analysis to resolve the cellular and molecular properties of TLS in the context of their spatial organization. To overcome this, we performed histology-guided laser microdissection of SjS SG foci defined by differing degrees of organization, using T/B cell area segregation and presence of fully formed CD21+GC as a proxy for evolving TLS^1^ (supplementary Fig. 6). Bulk sequencing and transcriptomic analysis were performed on isolated mRNA from non-segregated foci, segregated foci and foci with the formation of GC (TLS-GC). Tonsil germinal centers from healthy donors (tonsil-GC) were used as controls. PCA demonstrated sample clustering by level of maturation of the foci (Fig. 5a). To aid analysis we visualized expression of select genes known to be involved in lymphoneogenesis, co-stimulation, B cell apoptosis and survival (Fig. 5b). Enrichment of genes involved in inflammatory pathways, inflammatory cell recruitment and co-stimulation was observed in foci displaying progressive degrees of organization. Interestingly, transcripts from organized foci and GC^+^ TLS overlapped with some of the key genes defining tonsil-GC. However, segregated foci and TLS-GC also presented a strong inflammatory signature with increased transcripts for *ICOS*, *CXCL10*, *INFG*, *TNF*, *CASP8* and proinflammatory chemokine receptors (Fig. 5b). As expected, classical tonsil GC genes included lymphotoxin, *CXCL13*, *AICDA* and regulators of apoptosis, known to be critical for GC homeostasis.

We confirmed these differences between classical SLO and TLS at the protein level using a Nanostring Geomx proteomic panel, the targets of which largely overlapped with the genes interrogated in Fig. 5b. Analysis was performed in regions of interest defined by the same criteria used to select areas for microdissection and bulk RNA-sequencing. PCA based on expression data of 49 proteins broadly recapitulated the clustering observed in Fig. 5a with tonsil-GCs clustering separately and the remaining samples showing a progression in clustering from small foci to segregated foci into mature TLS-GCs (Fig. 5c). The heatmap in Fig. 5d illustrates clusters of proteins similar to those described for gene transcripts in Fig. 5b with CD127, OX40L, BCL, and Ki-67 forming a cluster with highest expression in Tonsil- GC. Protein expression for CD4, CD3, CD8, and CD68 displayed higher expression in all SG foci and TLS-GC than tonsil-GC along with higher expression of CD25, CD27, VISTA, and STING. Interestingly, higher expression of PD-1, PD-L1 and CD40 was detected in TLS-GC as compared to tonsil-GC (Fig. 5d).

**Fig. 6:**
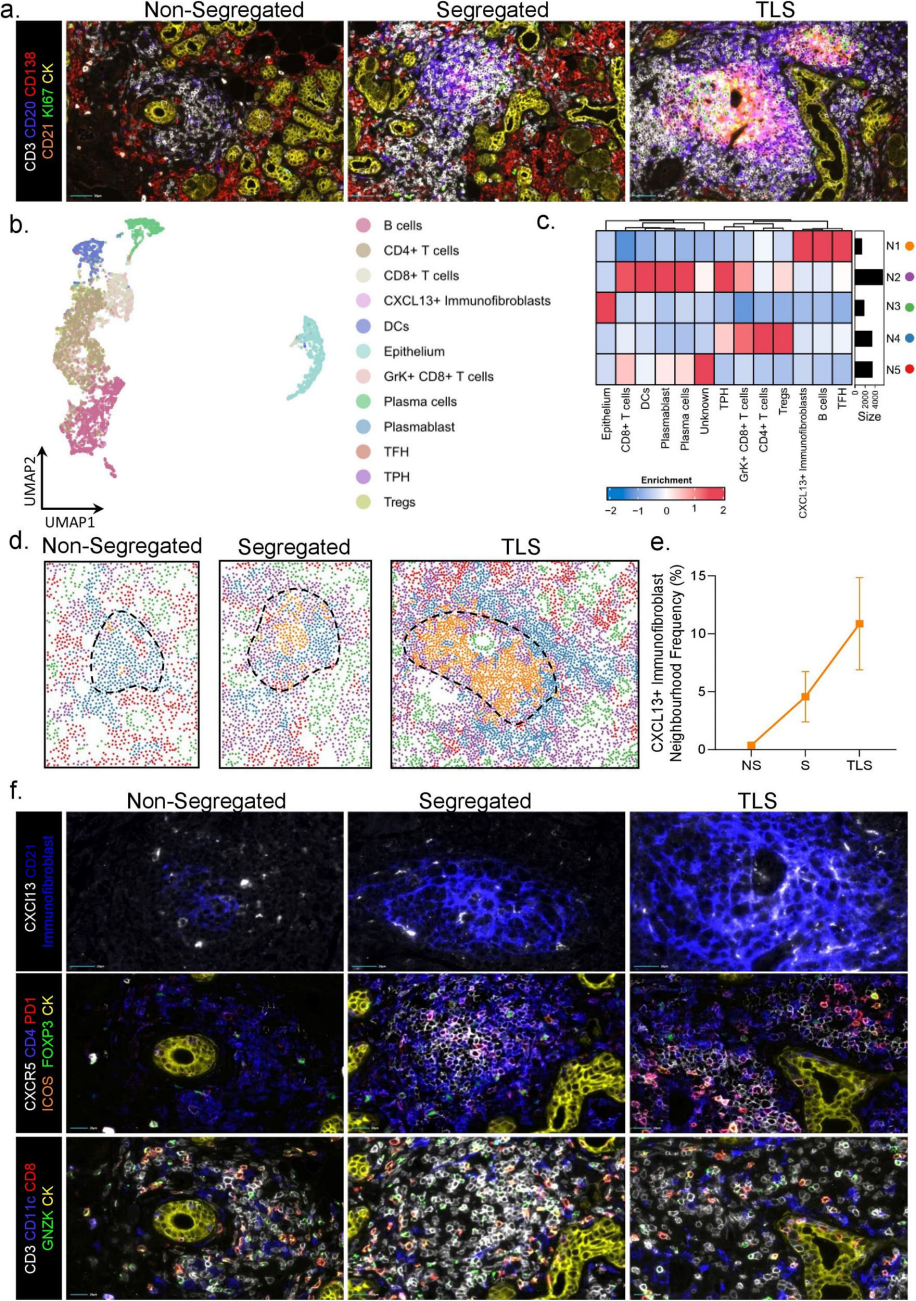
Single-cell spatial proteomics to identify spatially resolved cellular and structural landscape of TLS development. **a** Multiplex immunofluorescence image of salivary gland highlighting the development of immune aggregates (*n* = 2), Scale bar = 50 µm. **b** UMAP of single-cell proteomic phenotyping. **c** Cellular neighbourhood heatmap. **d** Spatial neighbourhood map (of a tertiary lymphoid structure (TLS)). **e** Abundance of neighbourhood 1 (CXCR5+B cell & Immunofibroblast enriched) as a function of developmental stage, data are representative as mean ± s.e.m, *n* = 2 per NS (non-segregated), S (segregated) and TLS developmental groups. **f** Multiplex immunofluorescence image of salivary gland (*n* = 2) highlighting key cell subsets across the development of immune aggregates, Scale bar = 20 µm.

Bulk mRNA analysis on microdissected TLS provided key additional information to our single cell analysis. However, this approach lacks the cellular resolution necessary to attribute specific cell populations to histologically distinct, evolving TLS. We therefore used Cibersortx to generate a signature expression matrix and estimate cell subset abundance in the bulk RNA-sequencing samples (Fig. 5e, supplementary Fig. 6b). As expected, estimated CCL19 TNFSF13B fibroblast cell proportions displayed a trend of increase from small foci to organized foci and TLS-GC samples. Interestingly, the potential precursors for this population (ACKR3^+^ cluster) appeared to be enriched in less mature TLS and progressively decrease with acquired organization and GC formation. Similarly, within the CD45^+^ leucocyte populations CD4 T follicular helper (T cell CD4 CXCL13) proportions progressively increased through the developmental stages of TLS with highest proportions in GC and tonsil samples. T helper 17-like (CCR6 LTB CD40LG T cells) estimated proportions followed a similar pattern, supporting the concept of a parallel development of both hematopoietic and stromal populations within TLS. To validate these data in tissue sections and define the spatially resolved cellular and structural components of TLS development, we performed hyperplex immunofluorescence imaging. Fully formed TLS and precursor (segregated & non-segregated) lymphoid aggregates were present throughout the tissue identifiable by prominent CD20^+^ B cell clusters and CD3^+^ T cell/CD20^+^ B cell interactions respectively (Fig. 6a). We defined the developmental stage of each structure as T/B segregated with GC, T/B segregated or non-segregated. Non-segregated structures showed variable B cell infiltration within a rich CD4^+^ T cell environment, whereas segregated structures showed a rich B cell core with minimal CXCR5 expression and sparse CD4^+^ T cell infiltrate within the core but a rich T cell border. Full TLS formation showed a bed of CXCR5^+^ B cells, with CD21 expression and CXCL13^+^ cells with a spindle morphology, suggestive of the TNFSF13B clusters (Fig. 6a–f). To quantify the differences in structure formation, the images were segmented to identify cell borders and the expression of each marker calculated for each cell. The expression profile of each cell identified 12 known phenotypes through single-cell clustering which were visualized on a UMAP and subsequently a spatial phenotype map (Fig. 6b, d). Given the importance of structural organization in these organs, we unbiasedly clustered tissue architectural features (cellular niches) using cellular neighborhoods to define 5 neighborhoods^44^. The enrichment of cells within these neighborhoods was visualized on a heatmap (Fig. 6c). Neighborhood 1 was enriched for CXCR5^+^ B cells, TFH and the TNFSF13B clusters representative of organized lymphoid structures. The abundance of neighborhood 1 increased with increasing TLS developmental stage, further defining, in our analysis, the ability to identify histological properties associated with full TLS maturation (Fig. 6e, f).

## Discussion

TLS are complex, evolving, multicellular structures able to shape the host immune response, impact disease trajectory and influence response to treatment in immune-mediated diseases and cancer^5,31,45,46^. Previous reports have applied in vitro and in vivo approaches to characterize the contribution of various cellular populations to TLS formation. However, these studies, performed before the use of single-cell analysis, did not exhaustively resolve TLS complex cellular composition, nor integrate these findings in the functional development of TLS^5,11,13^. In this study, we have integrated single-cell RNA sequencing, tissue transcriptomic and spatial proteomic data to deconvolute the cellular populations residing within human TLS, mapping spatial and functional interactions of TLS properties and their modulators as these structures evolve from small T cell aggregates to fully functional germinal centers.

We and others have previously identified fibroblasts as key elements for TLS establishment, as they precede and shape immune cell infiltration. In particular, we described a population of immunofibroblasts, stromal cells able to support lymphocyte migration and survival, while providing regulatory and structural function to the TLS^5^. The origin of this population was, however, not demonstrated in humans. In our dataset, we demonstrate the presence of a potential progenitor of the TLS-associated TNFSF13B clusters that is defined by the expression of *ACKR3* and *CD55* and shares key features with the PI16 universal fibroblast progenitor described by ref. ^21^. In order to evaluate the distribution of this cluster, and other clusters identified in our analysis during TLS development in humans, we performed CibersortX analysis on bulk RNA isolated and sequenced from microdissected SG-TLS. We used as proxy of developing TLS, lymphoid aggregates present in the SGs and characterized by different degree of organization, as those have been previously described as being reflective of the process of TLS maturation in both humans and animal model of TLS^5,11–13,16,47^. Interestingly, the transcripts encoded by our identified potential progenitor cluster appeared to progressively decrease in increasingly organized TLS; whilst transcripts associated with more specialized fibroblasts increased in those aggregates characterized by clear T/B cell segregation and formation of mature GCs. The phenomenon of fibroblast differentiation included acquisition of genes involved in the non-redundant functions of collagen metabolism and regulation of angiogenesis^14,48^ establishing within developing TLS functional microdomains to support cell-to-cell interaction, chemokine gradients to establish cell migration pathways and immunological functions.

The ability to produce lymphoid chemokines CCL19 and CCL21 has been previously associated with the differentiation of a specialized population of fibroblasts with immunoregulatory properties, defined as immunofibroblasts^5^. Here we confirmed the presence of this cluster in adult SjS SG single cells and reported increased abundance of this population was detected in largely organized TLS and TLS harboring functional germinal centers (GC+TLS). Importantly, the same population of immunofibroblasts was not identified in samples from patients with Sicca syndrome, a salivary gland disease, not characterized by TLS establishment. In our dataset we identified key differences in the properties specific to Sicca or SjS disease; in particular the enrichment in SjS, but not in Sicca, of specific cell populations (CXCL13 expressing T cells, LTB expressing T cells, and increased B cell and plasma cell state diversity)^12^. Moreover, we observed key variances in the differentiation of the fibroblast compartment, leading only in SjS and not in Sicca to the formation of a mature network of fibroblasts with immunological function. From our analysis it is not possible to establish whether these differences in fibroblast differentiation are due to differential imprinting in the common fibroblast progenitor or generated by the diverse microenvironmental cues present in the SG of patients with SjS and Sicca. In other autoimmune conditions, also characterized by TLS formation, production of autoantibodies has been reported to occur years prior to the establishment of tissue inflammation^12,14^. The same autoantibodies have been involved in direct activation of non-immune cells^12^, suggesting the intriguing possibility that autoantibodies might condition fibroblasts to acquire a specific phenotype, able to support TLS formation before the immune cells infiltrate the tissue. Animal data generated in rag^–/–^ mice appear to support this hypothesis, demonstrating early priming of fibroblasts in the absence of activated lymphocytes in tissue harboring TLS^5^. Receptor/ligand interaction analysis in our dataset further support these observations, suggesting disease specific interactions in SjS and Sicca that result in diverse microenvironments, divergent cluster maturation and anatomical signatures.

Additional immunoregulatory properties could be ascribed to the immunologically active pericytes, a small population of mural cells that we identified in our dataset. These are differentiated from other pericytes by expression of *CCL21* and *CCL19*, two chemokines instrumental for naïve T cell recruitment and T cell zone organization in SLOs^11,49,50^. CCL21^+^ CCL19^+^ pericytes do not express either LTβR or the genes classically associated with the alternative NFkB pathway, largely responsive for lymphoid chemokine expression^49,51^. In our dataset, this cluster was instead defined by genes involved in the response to TNFα and IFNγ, two cytokines highly upregulated in SjS. Recent reports suggest that pericytes can be precursors for and generate FSP1-expressing fibroblasts in aging mice but not in young mice^51^. In our dataset this was not demonstrated and, on the contrary, trajectory analysis appears to suggest a divergent differentiation pathway for the immunofibroblasts and the CCL21 CCL19 pericytes. This is in line with our previous findings in mice that reported on the fibroblast progenitor, the expression of PDGF-Ra, a receptor classically expressed only a small population of pericytes, likely associated with tissue remodeling^52^. Interestingly, the presence of CCL21 CCL19 pericytes precedes expansion of immunologically active fibroblasts as Cibersortx analysis suggested that pericytes able to secrete CCL21 and CCL19 are present with similar abundance in small foci and organized or GC^+^ TLS. Moreover, those are present in Sicca syndrome; however, in this condition, the cluster presents low expression of *CCL19*, most likely reflecting the decreased level of inflammation and lower transcripts of TNFα and IFNγ in Sicca. Given the totality of these data and the complexity of the cellular interactions unveiled by our bioinformatic analysis, we could speculate that, even in early inflammatory conditions, the differentiation of CCL21 CCL19 pericytes supports the formation of a functional unit for the early migration of activated T lymphocytes.

When first describing SLO formation, Ansel et al. reported the establishment of an amplificatory loop involving chemokines and lymphotoxin as a key element for the formation of the lymph node anlagen^53^. We have previously demonstrated that accumulation of activated T lymphocytes through the engagement of ICOS/ICOSL interaction results in local production of lymphotoxin and TNFα resulting in the initiation of the differentiation of chemokine expressing fibroblasts from resident progenitor cells. This inflammatory loop, once established becomes responsible for further recruitment of immune cells, including T follicular helper cells and B cells, providing not only spatial cues for cellular interaction, via chemokine production, but also survival factors and costimulation to incoming activated immune cells^11,25,35,54^.

Given the similarities between TLS and SLO we expected the profile of highly organized TLS harboring GC to mimic the gene profile of classical SLOs. In contrast, transcriptomics and proteomic analysis demonstrated clear differences between ectopic and physiological lymphoid structures and confirmed enrichment in certain cell types in mature TLS^8^. Importantly both gene analysis and proteomics confirmed in progressively organized foci, enrichment of genes involved in inflammatory pathways, inflammatory cell recruitment and co-stimulation. While a certain level of overlap was observed between transcripts and proteins expressed in GC^+^ TLS and tonsil-GC, inflammation-associated genes and proteins were enriched in TLS as compared to tonsils. Similarly, differences were observed in the expression of pro-survival and inflammatory genes, in genes regulating apoptosis, and in *AICDA*, the gene regulating somatic hypermutation and class switch recombination^55^. These differences suggest that TLS are highly inflammatory hubs potentially characterized by inadequate mechanisms of control, as low *AICDA* has been associated with poor clone selection in the GCs. The absence of this signal, in association with abundance of survival factors (*TNFSF13B*, *TNFSF14*), inflammatory cytokines and chemokines (*CXCL9*, *CXCL12*) and costimulatory molecules (*ICOS*), unveiled by our analysis could explain the occurrence of malignant clone escape that has been described TLS and is responsible for the increased frequency in development of mucosal associated lymphoid tissue lymphoma observed in SjS.

Taken together, these data provided us with important insights in the cellular and spatial complexity of TLS and the unique opportunity to compare the development of key cellular elements in two diseases characterized by different pathogenic pathways in the same target organ. This has enabled us to define a trajectory of development of the non-hematopoietic, stromal cells during TLS development and to map the interactions of these populations with incoming lymphocytes in progressive stages of TLS assembly, allowing us to share the first comprehensive integrated analysis of autoimmune TLS development in humans. The importance of TLS in the context of non-autoimmune disease is just starting to be appreciated, as prognostic factors for disease progression and response to therapy, raising the need for a better understanding of signals and pathways involved in TLS establishment and maintenance in the tissue. We believe that the identification of TLS cellular and molecular properties and their relevant modulators provided by our analysis can be of importance for other diseases characterized by TLS formation and provide therapeutic cues for multiple conditions.

## Methods

### Human samples

Labial minor salivary gland samples were obtained from patients recruited in the Optimising Assessment in Sjögren’s Syndrome (OASIS) cohort which recruits new patients attending the multidisciplinary Sjögren’s clinic at the Queen Elizabeth Hospital Birmingham, UK for assessment. SjS patients had a physician diagnosis of primary Sjögren’s syndrome and fulfilled the 2016 ACR/EULAR classification criteria. Participants with non-Sjögren’s sicca syndrome had signs and/or symptoms of dryness but did not have a physician diagnosis of SS or fulfill 2016 classification criteria. Salivary gland biopsy samples were divided in two: one for the scRNAseq study and the second for histological analysis to confirm diagnosis. Histological diagnosis is reported as presence of focal lymphocytic sialadenitis (FLS, suggestive of Primary Sjögren’s Syndrome, PSS) or non-specific chronic sialadenitis (NSCS), in the case of non-Sjögren’s sicca syndrome. All OASIS participants provided written informed consent, and the study was approved by the Wales Research Ethics Committee 7 (WREC 7) formerly Dyfed Powys REC; 13/WA/0392.

### Mice and salivary gland cannulation

C57BL/6 wild-type *(wt)* mice were purchased from Charles River. *Ltbr*^*–/–*^ mice were provided by Jorge Caamano. All mice were maintained under specific pathogen-free conditions in the Biomedical Service Unit at the University of Birmingham according to Home Office and local ethics committee regulations (University of Birmingham), under license no. P4B291FAA. Under ketamine/domitor anesthesia, the submandibular glands of female C57BL/6 and knock-out mice (8–12 weeks old) were intra-ductally cannulated with 10^8^–10^9^ p.f.u. of luciferase-encoding replication-defective adenovirus (Adv5). Mice salivary glands were harvested at day 15 post cannulation, the time-point when TLS are fully formed^5,13,40^.

### Isolation of cells from salivary glands

Minor salivary gland biopsies were taken surgically from the lip and frozen in 1 mL of CryoStor® CS10 (StemCell Technologies) at −80 °C. For preparation of single-cell suspension, firstly the frozen tissue sample in Cryotube were quickly thawed in water bath at 37 °C and washed twice in pre-warmed 5%FBS RPMI media. For mice, salivary gland was harvested at day 15 post cannulation and placed in 1 ml of RPMI-1640 (with 2% (v/v) FCS) on ice. Once all salivary glands were collected, RPMI-1640 was removed and replaced with 2 ml enzyme mix (RMPI with 2% FCS, 0.8 mg/ml Dispase, 0.2 mg/ml Collagenase P and 0.1 mg/ml DNase I).

The salivary gland tissue was then enzymatically digested for isolation of single cells^5^. Salivary glands were cut into small pieces and tubes were incubated at 37 °C in a water bath, with magnetic stirrers. After 20 min, salivary glands fragments were very gently pipetted using a 1 ml pipette, to further disrupt the tissue and release most cells. The mixture was replaced in the water bath and large fragments were allowed to settle for 30 s, after which the enzyme mix was removed. 10 ml of ice-cold FACS-EDTA buffer (0.5% (w/v) BSA, 2mM EDTA in PBS) was added and centrifuged (1800 rpm, 4 min, 4 °C). After centrifugation, 2 ml of fresh enzyme mix was added to the digestion tube, the contents gently mixed using a 1 ml pipette, and incubated, with regular gentle mixing using a 1 ml pipette. After 10 min, the cells were mixed vigorously for 30 s using a 1 ml pipette. Fragments were again allowed to settle, the supernatant was removed and added to the previously spun cell pellet, and 2 ml of fresh enzyme mix was added to the digestion tube. The digestion mix was then vigorously mixed using a 1 ml pipette every 5 min until it was clear that all remaining salivary gland fragments were completely digested. Supernatants were centrifuged after each removal (1800 rpm, 4 min, 4 °C) until finally, each collection tube contained the entire cellular contents of the salivary gland. Cells were filtered through 70μm nylon mesh and counted using a hemocytometer.

Dead cells were removed using the EasySepTM Dead Cell Removal (Annexin V) kit from the digested samples following manufacturer’s instructions before proceeding for the scRNA sequencing using the 10x platform. Or single cell suspension from salivary glands were used for flow cytometry analysis.

### Single-cell RNA sequencing

Library preparation and sequencing was completed by Oxford Genomics Centre. Cell counts in each sample were quantified using the Bio-Rad TC20. 10,000 cells per sample were used to generate libraries with the 10x Genomics v3 Single Cell 3’ kit before sequencing to a minimum depth of 50,000 reads per cell using the NovaSeq 6000.

### Single-cell sequencing alignment and pre-processing

Reads for each sample were aligned to the hg38 transcriptome and quantified using Cellranger v3.0.2 on the University of Birmingham BlueBEAR High Performance Computing service. Potential doublets were identified and removed using Scrublet v0.2.1 prior to sample aggregation and UMI normalisation using scripts adapted from the Sansom lab repository (https://github.com/sansomlab). Cells with greater than 200 unique genes expressed of which less than 20% are mitochondrial genes were taken forward for analysis using R v3.6.1 and Seurat v.2.3.4^56^. SjS samples and Sicca syndrome samples were analysed independently from this point following parallel analysis pipelines. Data were log normalized, the effects of the number of UMIs and percentage mitochondrial genes were regressed out before scaling and centering the data. For the comparative analysis of SjS and Sicca syndrome single-cell datasets, seurat objects were merged and renormalised and scaled.

### Cluster identification

Variable genes were identified by selecting outliers on a mean variability plot (Seurat FindVariableFeatures). Principal component analysis was completed before using Harmony v1.0 to minimise the effect of donor on subsequent clustering. Dimensional reduction was completed using the UMAP algorithm based on the harmony alignment. Clustering using the Seurat function FindClusters was investigated across resolutions from 0.2 to 1.2 and stability was assessed using Clustree v0.4.3. After gross cell types were identified the data was subset to each cell type and the process was iterated to identify subclusters. Final clustering was completed in R v4.0.3 and Seurat v4.0.2.

### Single-cell sequencing ambient background correction

To account for background noise with the single-cell sequencing dataset we implemented the probabilistic archetype assignment method from ref. ^22^. Cells with a larger probability of ambient noise than any other cell archetypes were removed from the dataset. The ambient probability for each cell was used in models for differential expression analysis during subclustering iterations.

### Differential expression analysis

Differential expression between cell clusters was completed using the Seurat function FindClusters with the MAST algorithm using the ambient probability as a latent variable within the model.

### Single-cell sequencing trajectory analysis

R v4.0.3 and slingshot v1.8 were used to run trajectory analysis on the fibroblast clusters in UMAP space.

### Gene set overrepresentation

Gene-set overrepresentation analysis of the fibroblast and mural subsets was run using gsfisher v0.2 (https://github.com/sansomlab). Features were selected for each cluster using the FindAllFeatures function (Seurat v4.0.3) running MAST with the calculated ambient probability as a latent variable. Enrichment was tested against GO terms and Kegg pathways.

### Single-cell sequencing ligand-receptor analysis

CellChat v1.4 was used to run ligand-receptor analysis on the single-cell sequencing dataset.

### Single-cell pseudobulk analysis

Single-cell RNA sequencing data were pseudobulked using the Seurat (v4.0.2) function AggregateExpression to sum counts by sample. Downstream analysis was then completed using DESeq2 (v1.30.1).

### Single-cell SCENIC regulon activity analysis

SCENIC v1.3.1 was used with default parameters to infer regulon activity within the single-cell RNA sequencing data.

### Healthy control salivary gland

Single cell RNA-sequencing data was obtained from ref. ^57^.

### Flow cytometry

Single cell suspensions were stained with Fixable Viability Dye eFluor™ 780 (65-0865-14; Invitrogen, 1:1000) in PBS as per manufacturer’s instructions. Following which cells were washed with FACS-EDTA buffer (PBS with 0.5% BSA and 2 mM EDTA) at 1800 rpm for 4 min and then incubated with antibodies on ice for 30 min in 100 μl FACS-EDTA buffer with ‘cocktails’ of the antibodies in Table 1 or Table 2.

**Table 1:** Human flow cytometry antibody panel

Marker	Clone	Isotype	Conjugate	Concentration (µl)	Supplier
CD36	5-271	Mouse IgG2a, κ	BV421	1/100	Biolegend
TNC	4C8MS	Mouse IgG1	AF700	1/100	Novus Biologicals
CD146	P1H12	Mouse IgG2a, κ	BV786	1/100	Biolegend
CD34	581	Mouse IgG1, κ	BV510	1/50	Biolegend
CD31	WM59	Rat IgG2a, κ	Alexa Fluor 488	1/200	Biolegend
EpCAM	9C4	Rat IgG2a, κ	Alexa Fluor 488	1/400	Biolegend
CD45	HI30	Rat / IgG2b, kappa	Alexa Fluor 488	1/300	Biolegend
CCL21	AF366	Polyclonal Goat IgG	Unconjugated	1/100	R&D Systems
Anti-Goat 2° Antibody	N/A	Donkey / IgG	PE	1/100	Invitrogen

**Table 2:** Mouse flow cytometry antibody panel

Marker	Clone	Isotype	Conjugate	Concentration (µl)	Supplier
CD34	Ram34	Rat / IgG2a, kappa	Alexa Fluor 700	1/100	Invitrogen
CD146	ME-9F1	Rat IgG2a		1/100	Biolegend
CD31	390	Rat IgG2a, κ	BV421	1/200	Biolegend
EpCAM	G8.8	Rat IgG2a, κ	BV605	1/400	Biolegend
CD45	30-F11	Rat / IgG2b, kappa	BV711	1/300	Biolegend
CCL21	AF457	Polyclonal Goat IgG	Unconjugated	1/50	R&D Systems
Anti-Goat 2°Antibody	N/A	Donkey / IgG	Alexa Fluor 647	1/100	Invitrogen

For intracellular staining, the cells were fixed in 50 µL of 2% PFA-PBS, pipetting for 30 s, and incubated on ice for 20 min. After fixation, 200 µL of 0.1% Saponin-PBS was added to each Eppendorf, and the cells were spun at 2200 rpm for 2 min before flicking off the liquid. The cells were then resuspended in 200 µL of 0.1% Saponin-PBS, spun again at 2200 rpm for 2 min, and the liquid was removed. For primary chemokine staining, the cells were incubated with 25 µL of CCL21 antibody in 0.1% Saponin-PBS on ice for 30 min. Following this, 200 µL of 0.1% Saponin-PBS was added, the cells were spun at 2200 rpm for 2 min, and the liquid was removed. This wash step was repeated. The cells were then stained with 25 µL of secondary antibody in 0.1% Saponin-PBS and incubated on ice for 30 min. Afterward, 200 µL of 0.1% Saponin-PBS was added, the cells were spun at 2200 rpm for 2 min, and the liquid was flicked off, repeating this wash step once more. Finally, the cells were resuspended in 400 µL of FACS-EDTA buffer were analyzed using BD LSR Fortessa flow cytometer. Data was analyzed with FlowJo.v10.

### Multiplex immunohistochemistry staining protocol and image acquisition

3 µm thick sections of FFPE tissue on super frost plus slides were sectioned. Indirect detection by fluorescence was based on the Opal^TM^ Multiplex IHC method (Akoya), performed on the Autostainer Leica Bond RX and Comet (Lunaphore). For staining in Comet, FFPE sectioned were deparaffinised and were subjected to a primary heat-induced epitope retrieval (HIER) in Tris EDTA buffer, pH 9.0 120 °C for 60 min in the PT-link (Lunaphore). Antibodies used were CD45 (Clone D9M8I, CST, 1:100), VE-cadherin (Clone E6N7A, CST, 1:100), Podoplanin/PDPN (EPR7072, Abcam, 1:100), CD90 (Clone D3V8A, CST, 1:100), CD146 (EPR3208; Abcam, 1:00), CD3 (Clone MRQ-39; Cell Marque, 1:500), CD20 (Clone L20; Cell Marque, 1:100), CD8 (Clone 4B11; Thermofisher, 1:50), CD4 (EPR6855; Abcam, 1:400), CD11c (Clone D3V1E; CST), PNAD (Clone MECA-79, Sigma-Aldrich, 1:50) CD82 (Clone D7G6H; CST, 1:200), Tenascin C (HPA004823; Sigma-Aldrich, 1:50), pan-cytokeratin (Clone AE1/AE3, Dako, 1:50), CD138 (Clone B-A38; Bio-Rad, 1:50), CD21 (Clone SP186; Abcam), Granzyme K (Clone 1C3A4; Proteintech, 1:250), FOXP3 (Clone SP97; Thermofisher, 1:50), ICOS (EPR20560; Abcam, 1:50), PD1 (EPR4877(2); Abcam, 1:500), CXCL13 (AF801; R&D systems, 1:20), CXCR5 (Clone D6L3C; CST, 1:25) and Ki67 (Clone MIB-1, Dako, 1:25). For the Akoya platform, immunofluorescent signal was visualized as per manufacturer’s instructions using the Opal 7-Color Automation IHC Kit (NEL871001KT, Akoya Biosciences) with TSA dyes 480, 520, 570, 620, 690 and 780 counterstained with Spectral DAPI. Slides were mounted with ProLong™ Diamond Antifade Mountant (P36965; Invitrogen) For the Lunaphore Comet, goat anti-mouse IgG Plus Alexa Fluor 555 (A32727; Invitrogen), goat anti-mouse IgM Alexa Fluor 555 (10143952; Fisher Scientific), goat anti-rabbit IgG Plus Alexa Fluor 647 (A32733; Invitrogen) and donkey anti-goat IgG Alexa Fluor 647 (A21447; Invitrogen). Multispectral images were acquired at ×20 magnification using the Vectra Polaris Automated Quantitative Pathology Imaging System (Akoya) or COMET (Lunaphore). MoTIF settings were used for multispectral image acquisition. Multispectral image processing of multiplex IHC stains was performed using Phenochart (version 1.0.11/Akoya) and inForm Image Analysis Software (version 2.3, Akoya) or Lunaphore image viewer.

### COMET multiplex IHC Image Processing and Cellular Segmentation

After acquisition, images were shading corrected, cycle aligned, stitched and merged into OME-TIFF pyramidal format within the Lunaphore COMET Explorer software. Autofluorescence for TRITC and Cy5 was conducted in the Lunaphore COMET Viewer v2. Single channels from each OME-TIFF stack were then extracted and regions containing lymphoid structures were sampled and saved as individual channel tiff files. Each region was segmented with CellSeg using default settings^58^, to identify each cell nucleus and membrane, and the segmentation mask for each was saved. These masks were then used to extract the expression, spatial and morphological information of each cell for the individual channel images. For nuclear channels (e.g., DAPI, Ki67 & FoxP3), the nuclear mask was used to extract cellular information. This information was saved as an FCS-like tabular data file.

### Cell Phenotype Identification and Neighbourhood Analysis

FCS-like files generated from COMET imaging were imported into R. Expression values were compressed to the 99.9th percentile per region. The morphology metrics cell size, solidity and eccentricity were z-scored and used as initial quality control steps to remove segmentation artefacts. Cell phenotyping was performed using CELESTA on each with a custom generated cell expression matrix^59^. The phenotype assignments were qualitatively validated by plotting them in their original spatial location and comparing the patterns to the images. The phenotypes were then visualized on a UMAP. Cells were assigned positive or negative for each functional protein by the findCutoff function of MetaCyto. Cellular neighborhoods were calculated by recording the 10 nearest neighbors of each cell and clustering these using k-Nearest Neighbors (kNN) with *k* = 6^44^.

### RNAscope

Indirect detection by fluorescence was based on the Opal^TM^ Multiplex IHC method (Akoya) and performed on the Autostainer Leica Bond RX. Samples were probed for RNAscope® LS 2.5 Probe - Hs-CCL19 (474369-C3), Hs-CCL21 (474378), Ms-CCL19 (432888) and Ms-CCL21a/b (432888-C3; 489928-C2) transcript variant 1 mRNA following manufacturer’s instructions (ACDBio). Subsequent antibody staining was performed with CD146 (EPR3208; Abcam, 1:00) in human salivary glands. Immunofluorescent signal was visualized using the Opal^TM^ 7-Color Automatic IHC Kit (Akoya) according to the manufacturer’s recommendations. Signal amplification and covalent binding of fluorophore was achieved by using a tyramide signaling amplification reagent (included in the Opal kit) that is conjugated with a different fluorophore for each cycle. Multispectral images were acquired at ×20 magnification using the Vectra Polaris Automated Quantitative Pathology Imaging System (Akoya). MoTIF settings were used for multispectral image acquisition. Multispectral image processing of multiplex IHC stains was performed using Phenochart (version 1.0.11/Akoya) and inForm Image Analysis Software (version 2.3, Akoya).

### RNA isolation, reverse transcription and quantitative PCR for mSGs from human SS patients

Total RNA from minor salivary gland biopsies of SjS patients and sicca syndrome was extracted using with a RNeasy mini kit (Qiagen) and the RNA was then reverse transcribed using the high-capacity reverse transcription cDNA synthesis kit (Applied Biosystems) according to the manufacturer’s specifications. Reverse transcription was carried out on Techne 312 Thermal Cycler PCR machine. Quantitative RT-PCR (Applied Biosystems) was performed on cDNA samples Quantitative real-time PCR was performed on a 7900HT Fast Real-Time PCR System (Applied biosystems) using TaqMan Gene Expression Master Mix (Applied biosystems) and the following primers: IFNG (Hs00989291_m1), CXCL9 (Hs00171065_m1), CCL21 (Hs99999110_m1), CD40 (Hs01002915_g1), CXCL13 (Hs00757930_m1), CCL19 (Hs00171149_m1), TNFSF13B (Hs00198106_m1) and TNFRSF13B (Hs00963364_m1). All samples were run in duplicate and replicates with more than 2 cycles between them were discarded. Gene expression data was analysed using the 2^-delta Ct method using GAPDH (Hs02758991_g1) as housekeeping gene.

### Microdissection and Bulk-RNA sequencing

The selected tissue samples for microdissection processing were acquired from patients recruited within the OASIS cohort at the University of Birmingham, under ethics number 10-018. Preparation of the samples for microdissection: all surfaces of the cryostat and lab bench were thoroughly cleaned with 70% Etoh and sterilised with ultraviolet (UV) light for at least 30 min. Containers used to keep store the collected samples and the filter paper used to separate slides (Membrane slides, Carl Zeiss™ 1.0 PEN NF 41590-9081-000) must also be treated with UV for 30 min and kept sterile before handling the tissue. To cut tissue for microdissection, the UV sterilised membrane slides were maintained at a cool temperature within the cryostat prior to sample cutting. The tissue was mounted on a cryostat block with as little mounting media (OCT, TissueTeK) as possible to minimise interference with laser cutter during microdissection. Sections were cut at 8 μm thickness and transferred to the membrane slides. Once the section had been mounted onto the membrane slides, the membrane slide was stored in its carrier box and in dry ice until final storage at –80 °C prior to staining with Cresyl violet acetate (SIGMA). Preparation of Cresyl violet acetate is required one day prior to staining the mounted tissues. Preparation of the CresylViolet (1% solution) included dissolving the powder in 80% ethanol which had been made using sterile RNase-free water in a MSC Class II ventilator system. This solution was mixed overnight on a shaker at 4 °C to ensure that the CresylViolet was completely dissolved, and the solution was then passed through a 70 µm strainer before use. The slides were first dipped in RNase-free water and suspended for 2 min in 70% Etoh, also prepared in RNase-free water, to facilitate the removal of OCT on the tissue. The slides were then suspended in the CresylViolet 1% solution for 30 s to 1 min and excess solution was tapped off. The slides were then processed for 1 min in 100% Etoh and this step was repeated three times. The PALM slides were then left to dry until the membrane was observed to be completely dry. The PALM slides were then stored at –80 °C in a parafilm sealed 50 ml falcon until the samples were ready for microdissection.

Prior to microdissection, the PALM slides were bought to room temperature for 10 min. Microdissection was completed using the PALM Robo Software V.4.6 software using the brightfield “AxioCam CC1” setting. The objective lens used to micro dissects SG sections was 10X whilst tonsil was set at 5X. The area of interest was then identified using the ocular eye piece. Once the region had been selected the laser was aligned to the section that was to be cut. The collection tubes required for the capture of the microdissected tissue contained 20 μl of RLT buffer with B-mercaptanol, ensuring the lid was kept dry, and tube was kept on dry ice prior to use. The collection tubes were then mounted onto the *Collector Set 200 CMII* and set to the correct position to collect the microdissected sample as they were cut by applying the calibration procedure to collect tissue in the center of the lid of the collection tube which were stored at –80 °C prior to RNA extraction. RNA extraction from the microdissected samples was completed as per the protocols provided using the RNeasy FFPE kit (Qiagen).

Library preparation and sequencing from extracted RNA was completed by Genomics Birmingham. Initial libraries were prepared using the SMART-Seq v4 Ultra Low Input RNA Kit. Further samples were processed using the NEBNext rRNA Depletion Kit v2 and NEBNext Ultra II Directional RNA Library Prep Kit for Illumina with NEBNext Multiplex Oligos for Illumina. All libraries were sequenced using a NextSeq 500 sequencer. Adapters were trimmed with trimmomatic v0.39, reads aligned to the hg38 transcriptome with STAR v2.7.2b, and counts summed using featureCounts in Subread v2.0.1 using the University of Birmingham BlueBEAR High Performance Computing service. R v4.0.3 and DESeq2 v1.30.1 were used for downstream analysis. To minimise batch effects during differential expression analysis batch was included as a factor in the DESeq2 model and for principal component analysis and visualizations batch effects were minimised in variance stabilising transformed data using the limma v3.46.0 function removeBatchEffect.

### Geomx nanostring

The Nanostring GeoMX Digital Spatial Profiler (DSP) Protein slide preparation was followed according to the manufacturer’s instruction (https://nanostring.com/products/geomx-digital-spatial-profiler/geomx-protein-assays). Briefly, the slides were baked, deparaffinized, and antigen retrieval was performed using Citrate buffer pH 6.0. Next, the samples were incubated with 84 oligo-labeled primary antibodies: a Human Immune Cell Profiling Core, a Human Immune Activation Status Panel, a Human Immune Cell Typing Panel, a Human Immune Cell Death Panel and a Human Immune Drug IO Panel. The same sections were then stained with fluorescently labeled SYTO13 (S7575; Invitrogen), CD45-Alexa Fluor 532 (D9M8I, CST, 1:100), CD21-PE/Dazzle 594 (Clone Bu32, Biolegend, 1:25) and CD138-Alexa Fluor 647 (Clone MI16, Biolegend, 1:25) to identify tissue regions of interest (ROI). The slides were then loaded onto the GeoMX DSP and 4-6 ROIs were randomly selected per slide and analyzed for protein expression. All the indexing oligonucleotides were collected into a 96-well plate and were then hybridized to fluorescent barcodes using GeoMX Hyb Codes. After hybridization, samples were processed using the using the nCounter system according to the manufacturer’s instructions. 49 regions of interest passed QC criteria and were taken forward for analysis. Using the Geomx software we scaled the data to number of nuclei/ROI and subsequently normalized using negative control probes. Data were exported and PCA analysis and heatmaps were generated using R v4.0.3 and the pheatmap v1.0.12 package.

### Cell type abundance estimation

Using the deconvolution tool Cibersortx^60^, the SjS single-cell data was used as a reference dataset to generate a signature matrix which was used to impute cell type abundance in the bulk RNA-sequencing samples. 100 permutations were run for the imputation.

### Statistics & reproducibility

No statistical method was used to predetermine sample size for this study. Only in single-cell RNA sequencing, one sicca and one SjS sample failed alignment and sequencing data QC and were excluded from further analysis, and a further sicca sample was excluded due to unusual clinical findings. Graphs were prepared for Figs. 3d, 4d and 6e and statistics in Figs. 3d and 4d were performed with Prism software (GraphPad software Inc, San Diego, CA) using a two-tailed Mann-Whitney test, student’s *t*-test.

### Reporting summary

Further information on research design is available in the Nature Portfolio Reporting Summary linked to this article.

## Supplementary information

Supplementary Information

Description of Additional Supplementary Files

Supplementary Data 1

Supplementary Data 2

Supplementary Data 3

Reporting Summary

## Source data

Source Data

## Data Availability

The Single-cell RNA sequencing data generated in this study have been deposited in the Gene Expression Omnibus database under accession code GSE272409. The Bulk RNA sequencing data generated in this study have been deposited in the Gene Expression Omnibus database under accession code GSE272410. Source data are provided with this paper.

## References

[1] Helmink, B. A. et al. B cells and tertiary lymphoid structures promote immunotherapy response. *Nature* **577**, 549–555 (2020). 31942075 10.1038/s41586-019-1922-8 PMC8762581

[2] Petitprez, F. et al. B cells are associated with survival and immunotherapy response in sarcoma. *Nature* **577**, 556–560 (2020). 31942077 10.1038/s41586-019-1906-8

[3] Goc, J., Fridman, W.-H., Sautès-Fridman, C. & Dieu-Nosjean, M.-C. Characteristics of tertiary lymphoid structures in primary cancers. *Oncoimmunology* **2**, e26836 (2013). 10.4161/onci.26836 PMC3912008 24498556

[4] Huibers, M. M. et al. The composition of ectopic lymphoid structures suggests involvement of a local immune response in cardiac allograft vasculopathy. *J. Heart Lung Transpl.* **34**, 734–745 (2015). 10.1016/j.healun.2014.11.022 25655346

[5] Nayar, S. et al. Immunofibroblasts are pivotal drivers of tertiary lymphoid structure formation and local pathology. *Proc. Natl Acad. Sci.* **116**, 13490–13497 (2019). 31213547 10.1073/pnas.1905301116 PMC6613169

[6] Lucchesi, D. & Bombardieri, M. The role of viruses in autoreactive B cell activation within tertiary lymphoid structures in autoimmune diseases. *J. Leukoc. Biol.* **94**, 1191–1199 (2013). 10.1189/jlb.0413240 23812327

[7] Neyt, K., Perros, F., GeurtsvanKessel, C. H., Hammad, H. & Lambrecht, B. N. Tertiary lymphoid organs in infection and autoimmunity. *Trends Immunol.* **33**, 297–305 (2012). 22622061 10.1016/j.it.2012.04.006 PMC7106385

[8] Cabrita, R. et al. Tertiary lymphoid structures improve immunotherapy and survival in melanoma. *Nature* **577**, 561–565 (2020). 31942071 10.1038/s41586-019-1914-8

[9] Colbeck, E. J., Ager, A., Gallimore, A. & Jones, G. W. Tertiary lymphoid structures in cancer: drivers of antitumor immunity, immunosuppression, or bystander sentinels in disease? *Front. Immunol.* **8**, 1830-1830 (2017). 10.3389/fimmu.2017.01830 PMC5742143 29312327

[10] Asam, S., Nayar, S., Gardner, D. & Barone, F. Stromal cells in tertiary lymphoid structures: architects of autoimmunity. *Immunol. Rev.* **302**, 184–195 (2021). 10.1111/imr.12987 34060101

[11] Barone, F. et al. Association of CXCL13 and CCL21 expression with the progressive organization of lymphoid-like structures in Sjogren’s syndrome. *Arthritis Rheum.* **52**, 1773–1784 (2005). 15934082 10.1002/art.21062

[12] Barone, F. et al. IgA-Producing plasma cells originate from germinal centers that are induced by B-cell receptor engagement in humans. *Gastroenterology* **140**, 947–956 (2011). 21147106 10.1053/j.gastro.2010.12.005 PMC7115992

[13] Barone, F. et al. IL-22 regulates lymphoid chemokine production and assembly of tertiary lymphoid organs. *Proc. Natl Acad. Sci.* **112**, 11024–11029 (2015). 26286991 10.1073/pnas.1503315112 PMC4568258

[14] Wahren, M., Solomin, L., Pettersson, I. & Isenberg, D. Autoantibody repertoire to Ro/SSA and La/SSB antigens in patients with primary and secondary Sjögren’s syndrome. *J. Autoimmun.* **9**, 537–544 (1996). 8864830 10.1006/jaut.1996.0072

[15] Salomonsson, S. et al. Cellular basis of ectopic germinal center formation and autoantibody production in the target organ of patients with Sjogren’s syndrome. *Arthritis Rheum.* **48**, 3187–3201 (2003). 14613282 10.1002/art.11311

[16] Barone, F. et al. CXCL13, CCL21, and CXCL12 expression in salivary glands of patients with Sjögren’s Syndrome and MALT lymphoma: association with reactive and malignant areas of lymphoid organization. *J. Immunol.* **180**, 5130–5140 (2008). 18354239 10.4049/jimmunol.180.7.5130

[17] Alunno, A., Leone, M. C., Giacomelli, R., Gerli, R. & Carubbi, F. Lymphoma and lymphomagenesis in primary Sjögren’s syndrome. *Front. Med.* **5**, 102-102 (2018). 10.3389/fmed.2018.00102 PMC5909032 29707540

[18] Mueller, C. G., Nayar, S., Gardner, D. & Barone, F. Cellular and vascular components of tertiary lymphoid structures. *Methods Mol. Biol.* **1845**, 17–30 (2018). 30141005 10.1007/978-1-4939-8709-2_2

[19] Benezech, C. et al. Inflammation-induced formation of fat-associated lymphoid clusters. *Nat. Immunol.* **16**, 819–828 (2015). 26147686 10.1038/ni.3215 PMC4512620

[20] Korsunsky, I. et al. Cross-tissue, single-cell stromal atlas identifies shared pathological fibroblast phenotypes in four chronic inflammatory diseases. *Medicine* **3**, 481–518.e14 (2022). 10.1016/j.medj.2022.05.002 PMC9271637 35649411

[21] Buechler, M. B. et al. Cross-tissue organization of the fibroblast lineage. *Nature* **593**, 575–579 (2021). 33981032 10.1038/s41586-021-03549-5

[22] Wei, K. et al. Notch signalling drives synovial fibroblast identity and arthritis pathology. *Nature* **582**, 259–264 (2020). 10.1038/s41586-020-2222-z PMC7841716 32499639

[23] Davidson, S. et al. Single-cell RNA sequencing reveals a dynamic stromal niche that supports tumor growth. *Cell Rep.* **31**, 107628 (2020). 32433953 10.1016/j.celrep.2020.107628 PMC7242909

[24] Rodda, L. B. et al. Single-cell RNA sequencing of lymph node stromal cells reveals niche-associated heterogeneity. *Immunity* **48**, 1014–1028.e1016 (2018). 29752062 10.1016/j.immuni.2018.04.006 PMC5971117

[25] Link, A. et al. Association of T-zone reticular networks and conduits with ectopic lymphoid tissues in mice and humans. *Am. J. Pathol.* **178**, 1662–1675 (2011). 21435450 10.1016/j.ajpath.2010.12.039 PMC3070229

[26] Kumar, V. et al. A dendritic-cell-stromal axis maintains immune responses in lymph nodes. *Immunity* **42**, 719–730 (2015). 25902483 10.1016/j.immuni.2015.03.015 PMC4591553

[27] Gregory, J. L. et al. Infection programs sustained lymphoid stromal cell responses and shapes lymph node remodeling upon secondary challenge. *Cell Rep.* **18**, 406–418 (2017). 28076785 10.1016/j.celrep.2016.12.038

[28] Fletcher, A. L. et al. Lymph node fibroblastic reticular cells directly present peripheral tissue antigen under steady-state and inflammatory conditions. *J. Exp. Med.* **207**, 689–697 (2010). 20308362 10.1084/jem.20092642 PMC2856033

[29] Fletcher, A. L., Acton, S. E. & Knoblich, K. Lymph node fibroblastic reticular cells in health and disease. *Nat. Rev. Immunol.* **15**, 350–361 (2015). 25998961 10.1038/nri3846 PMC5152733

[30] Krishnamurty, A. T. & Turley, S. J. Lymph node stromal cells: cartographers of the immune system. *Nat. Immunol.* **21**, 369–380 (2020). 32205888 10.1038/s41590-020-0635-3

[31] Barone, F. et al. Stromal fibroblasts in tertiary lymphoid structures: a novel target in chronic inflammation. *Front. Immunol.* **7**, 477 (2016). 10.3389/fimmu.2016.00477 PMC5100680 27877173

[32] Bartoschek, M. et al. Spatially and functionally distinct subclasses of breast cancer-associated fibroblasts revealed by single cell RNA sequencing. *Nat. Commun.* **9**, 5150 (2018). 30514914 10.1038/s41467-018-07582-3 PMC6279758

[33] Chen, Z. et al. Single-cell RNA sequencing highlights the role of inflammatory cancer-associated fibroblasts in bladder urothelial carcinoma. *Nat. Commun.* **11**, 5077 (2020). 33033240 10.1038/s41467-020-18916-5 PMC7545162

[34] Croft, A. P. et al. Distinct fibroblast subsets drive inflammation and damage in arthritis. *Nature* **570**, 246–251 (2019). 31142839 10.1038/s41586-019-1263-7 PMC6690841

[35] Pipi, E. et al. Tertiary lymphoid structures: autoimmunity goes local. *Front. Immunol.* **9**, 1952 (2018). 10.3389/fimmu.2018.01952 PMC6143705 30258435

[36] García-Cuesta, E. M. et al. The role of the CXCL12/CXCR4/ACKR3 axis in autoimmune diseases. *Front. Endocrinol.* **10**, 585 (2019). 10.3389/fendo.2019.00585 PMC6718456 31507535

[37] Ji, Z., He, L., Regev, A. & Struhl, K. Inflammatory regulatory network mediated by the joint action of NF-kB, STAT3, and AP-1 factors is involved in many human cancers. *Proc. Natl Acad. Sci.* **116**, 9453–9462 (2019). 30910960 10.1073/pnas.1821068116 PMC6511065

[38] Vondenhoff, M. F. et al. LTbetaR signaling induces cytokine expression and up-regulates lymphangiogenic factors in lymph node anlagen. *J. Immunol.* **182**, 5439–5445 (2009). 19380791 10.4049/jimmunol.0801165 PMC2703443

[39] Bénézech, C. et al. Lymphotoxin-β receptor signaling through NF-κB2-RelB pathway reprograms adipocyte precursors as lymph node stromal cells. *Immunity* **37**, 721–734 (2012). 22940098 10.1016/j.immuni.2012.06.010 PMC3809035

[40] Bombardieri, M. et al. Inducible tertiary lymphoid structures, autoimmunity, and exocrine dysfunction in a novel model of salivary gland inflammation in C57BL/6 mice. *J. Immunol.* **189**, 3767–3776 (2012). 22942425 10.4049/jimmunol.1201216 PMC3448973

[41] Loureiro-Amigo, J. et al. Utility of lymphocyte phenotype profile to differentiate primary Sjögren’s syndrome from sicca syndrome. *Rheumatology* **60**, 5647–5658 (2021). 33620072 10.1093/rheumatology/keab170

[42] Fisher, B. A. et al. Standardisation of labial salivary gland histopathology in clinical trials in primary Sjögren’s syndrome. *Ann. Rheum. Dis.* **76**, 1161–1168 (2017). 27965259 10.1136/annrheumdis-2016-210448 PMC5530351

[43] Pucino, V., Gardner, D. H. & Fisher, B. A. Rationale for CD40 pathway blockade in autoimmune rheumatic disorders. *Lancet Rheumatol.* **2**, e292–e301 (2020). 38273474 10.1016/S2665-9913(20)30038-2

[44] Schürch, C. M. et al. Coordinated cellular neighborhoods orchestrate antitumoral immunity at the colorectal cancer invasive. *Front. Cell* **182**, 1341–1359.e1319 (2020). 32763154 10.1016/j.cell.2020.07.005 PMC7479520

[45] Sautès-Fridman, C., Petitprez, F., Calderaro, J. & Fridman, W. H. Tertiary lymphoid structures in the era of cancer immunotherapy. *Nat. Rev. Cancer* **19**, 307–325 (2019). 31092904 10.1038/s41568-019-0144-6

[46] Teillaud, J. L., Regard, L., Martin, C., Sibéril, S. & Burgel, P. R. Exploring the role of tertiary lymphoid structures using a mouse model of bacteria-infected lungs. *Methods Mol. Biol.* **1845**, 223–239 (2018). 30141016 10.1007/978-1-4939-8709-2_13

[47] Nayar, S. et al. Bimodal expansion of the lymphatic vessels is regulated by the sequential expression of IL-7 and lymphotoxin α1β2 in newly formed tertiary lymphoid structures. *J. Immunol.* **197**, 1957–1967 (2016). 27474071 10.4049/jimmunol.1500686 PMC4991245

[48] Nocturne, G. & Mariette, X. Sjögren syndrome-associated lymphomas: an update on pathogenesis and management. *Br. J. Haematol.* **168**, 317–327 (2015). 25316606 10.1111/bjh.13192

[49] Ager, A. High endothelial venules and other blood vessels: critical regulators of lymphoid organ development and function. *Front. Immunol.* **8**, 45 (2017). 10.3389/fimmu.2017.00045 PMC5289948 28217126

[50] Veerman, K., Tardiveau, C., Martins, F., Coudert, J. & Girard, J.-P. Single-cell analysis reveals heterogeneity of high endothelial venules and different regulation of genes controlling lymphocyte entry to lymph nodes. *Cell Rep.* **26**, 3116–3131.e3115 (2019). 30865898 10.1016/j.celrep.2019.02.042

[51] Chen, J. et al. High-resolution 3D imaging uncovers organ-specific vascular control of tissue aging. *Sci. Adv.* **7**, eabd7819 (2021). 10.1126/sciadv.abd7819 PMC7857692 33536212

[52] Wilson, C. L. et al. Characterization of human PDGFR-β-positive pericytes from IPF and non-IPF lungs. *Am. J. Physiol. Lung Cell Mol. Physiol.* **315**, L991–L1002 (2018). 30335500 10.1152/ajplung.00289.2018 PMC6337011

[53] Ansel, K. M. et al. A chemokine-driven positive feedback loop organizes lymphoid follicles. *Nature* **406**, 309–314 (2000). 10917533 10.1038/35018581

[54] Pikor, N. B. et al. Remodeling of light and dark zone follicular dendritic cells governs germinal center responses. *Nat. Immunol.* **21**, 649–659 (2020). 32424359 10.1038/s41590-020-0672-y PMC7610477

[55] Bombardieri, M. et al. Activation-induced cytidine deaminase expression in follicular dendritic cell networks and interfollicular large B cells supports functionality of ectopic lymphoid neogenesis in autoimmune sialoadenitis and MALT lymphoma in Sjögren’s syndrome. *J. Immunol.* **179**, 4929–4938 (2007). 10.4049/jimmunol.179.7.4929 17878393

[56] Hao, Y. et al. Integrated analysis of multimodal single-cell data. *Cell* **184**, 3573–3587.e3529 (2021). 34062119 10.1016/j.cell.2021.04.048 PMC8238499

[57] Huang, N. et al. SARS-CoV-2 infection of the oral cavity and saliva. *Nat. Med.* **27**, 892–903 (2021). 33767405 10.1038/s41591-021-01296-8 PMC8240394

[58] Lee, M. Y. et al. CellSeg: a robust, pre-trained nucleus segmentation and pixel quantification software for highly multiplexed fluorescence images. *BMC Bioinform.* **23**, 46 (2022). 10.1186/s12859-022-04570-9 PMC8767664 35042474

[59] Zhang, W. et al. Identification of cell types in multiplexed in situ images by combining protein expression and spatial information using CELESTA. *Nat. Methods* **19**, 759–769 (2022). 35654951 10.1038/s41592-022-01498-z PMC9728133

[60] Newman, A. M. et al. Determining cell type abundance and expression from bulk tissues with digital cytometry. *Nat. Biotechnol.* **37**, 773–782 (2019). 31061481 10.1038/s41587-019-0114-2 PMC6610714

