# Supplementary material for: Molecular and spatial analysis of tertiary lymphoid structures in Sjogren’s syndrome
Source: Nat Commun. 2025 Jan 2;16:5. doi: 10.1038/s41467-024-54686-0 (PMC11697438; doi:10.1038/s41467-024-54686-0)
Supplement: Supplementary file 1 — Supplementary Information [file 41467_2024_54686_MOESM1_ESM.pdf]

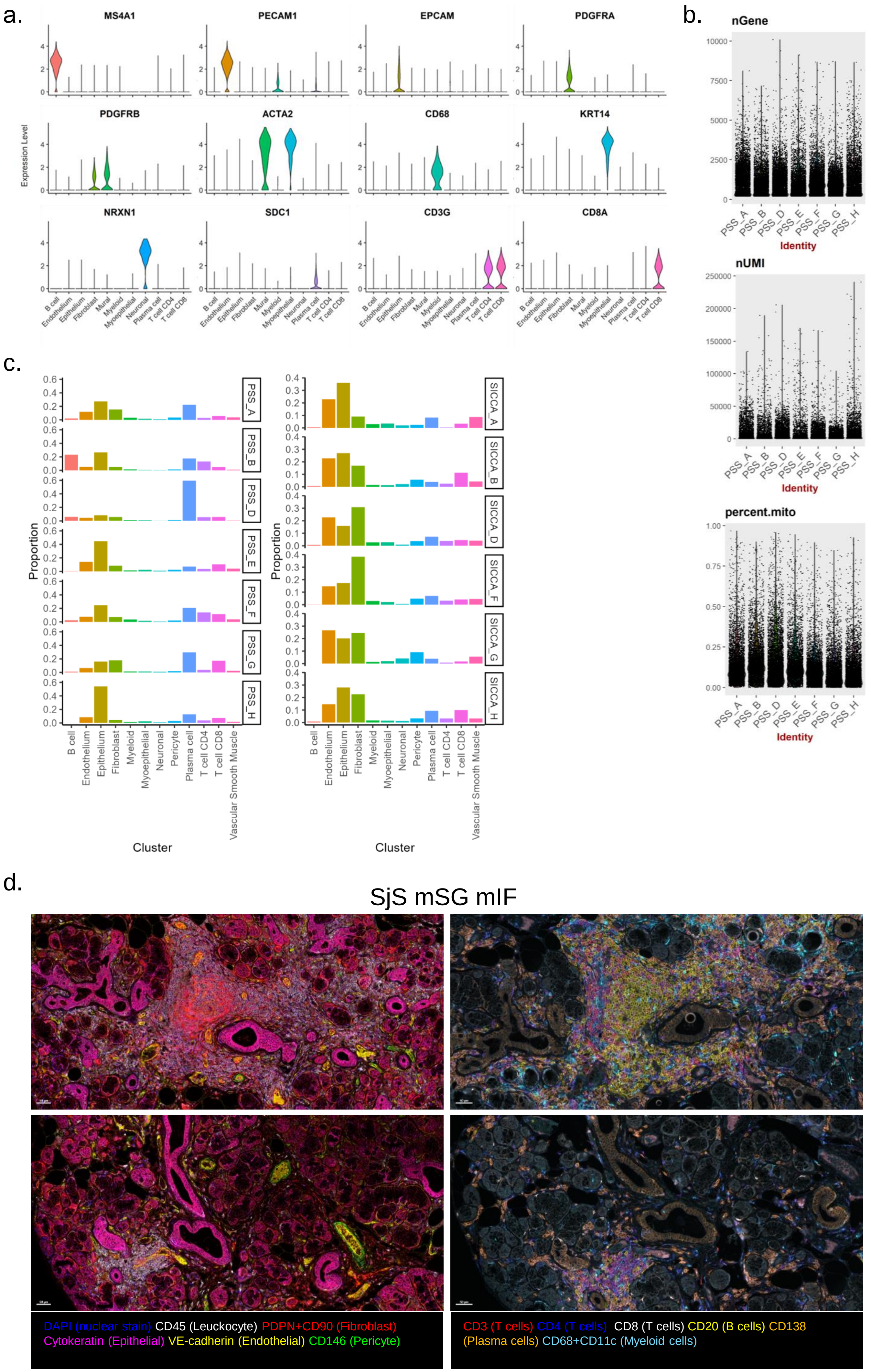

Supplementary figure 1. a, Expression of marker genes identifying gross-cell identities isolated from Sjogren's minor salivary glands. **b,** Plots of the number of genes, number of UMIs, and percentage mitochondrial genes per sample. **c,** Proportions of gross cell states across samples. **d,** Multiplex immunofluorescence image illustrating major lineages identified in minor salivary gland tissue from Sjogren's patients probed with a 6-plex panels for CD146 (green), VE-cadherin or CD20 (yellow), Pan-Cytokeratin/CK (magenta), CD138 (orange), and CD68+CD11c (cyan), CD45 or CD8 (white), Podoplanin/PDPN+CD90 or CD3 (red) and CD4 (blue) , Scale bar=50µm.

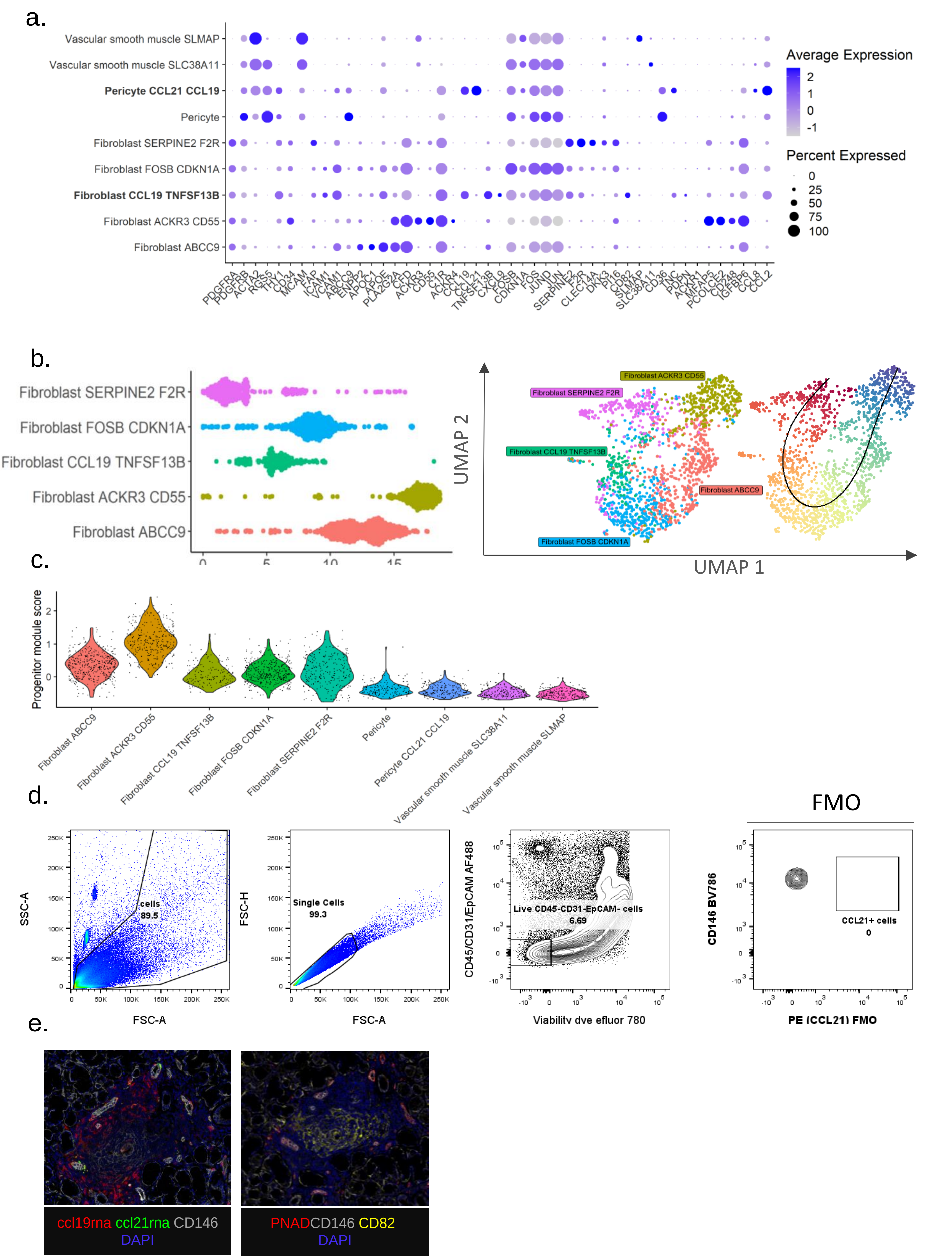

Supplementary figure 2. a, Expanded dotplot of previous and newly identified genes differentiating between fibroblast and mural clusters. **b,** Slingshot trajectory analysis of fibroblast clusters plotted against pseudotime or mapped to UMAP coordinates. **c,** Progenitor module score across fibroblast and mural populations generated using the Seurat function AddModuleScore with features identified as marking a progenitor population in the Buechler *et al* [21] dataset (specifically the genes *ACKR3*, *PI16*, *MFAP5*, *PCOLCE2*, *C3*, *PLA2G2A*, *IGFBP6*, *CD248*). **d,** CCL21 FMO control of flow cytometric identification of CCL21 CCL19 pericytes in human salivary glands. **e,** Multiplex immunofluorescence image identifying the *ccl21rna+ccl19rna+*CD146+ pericyte associates vessels within TLS in minor salivary gland tissue probed with a CD146 (grey), CD82 (yellow), PNAD (red) and DAPI (blue), Scale bar=50µm.

a.

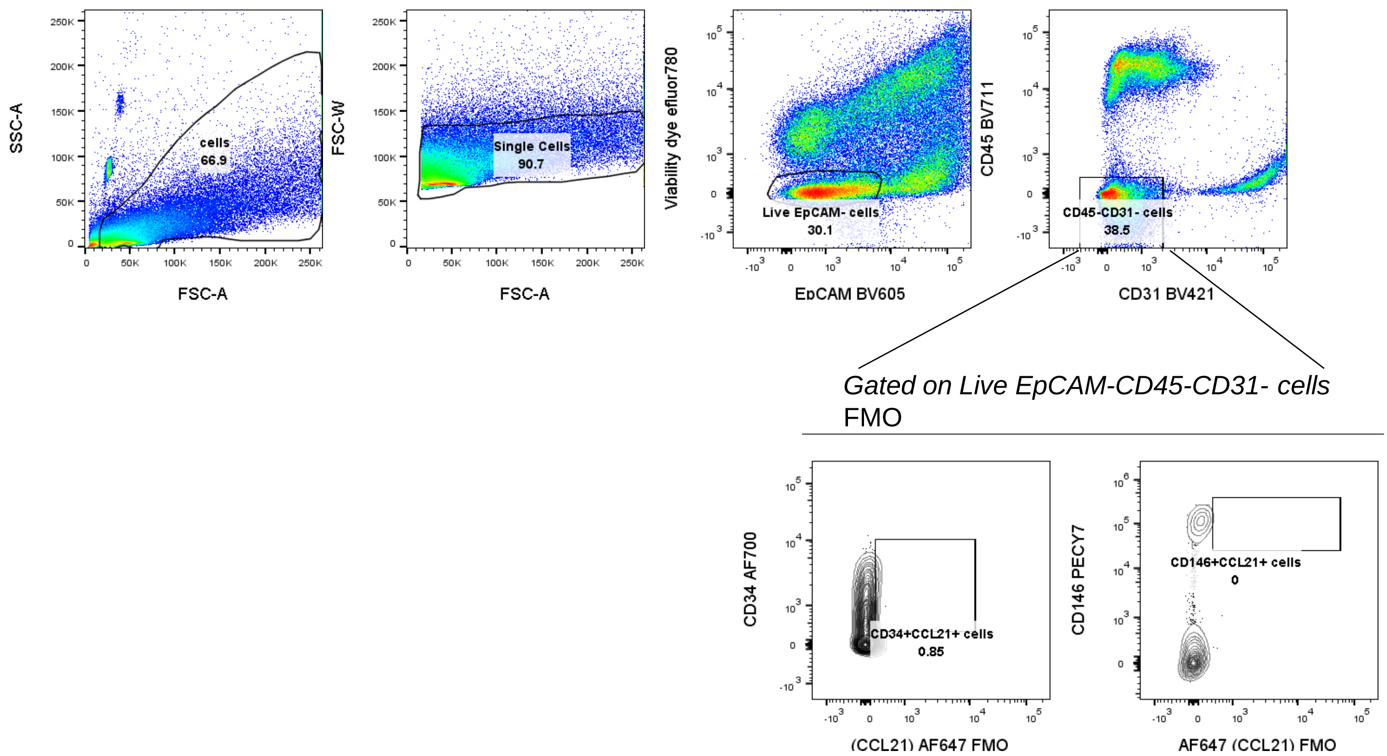

Supplementary figure 3. a, CCL21 FMO control of flow cytometric identification of CCL21 CCL19 pericytes in mouse salivary glands.

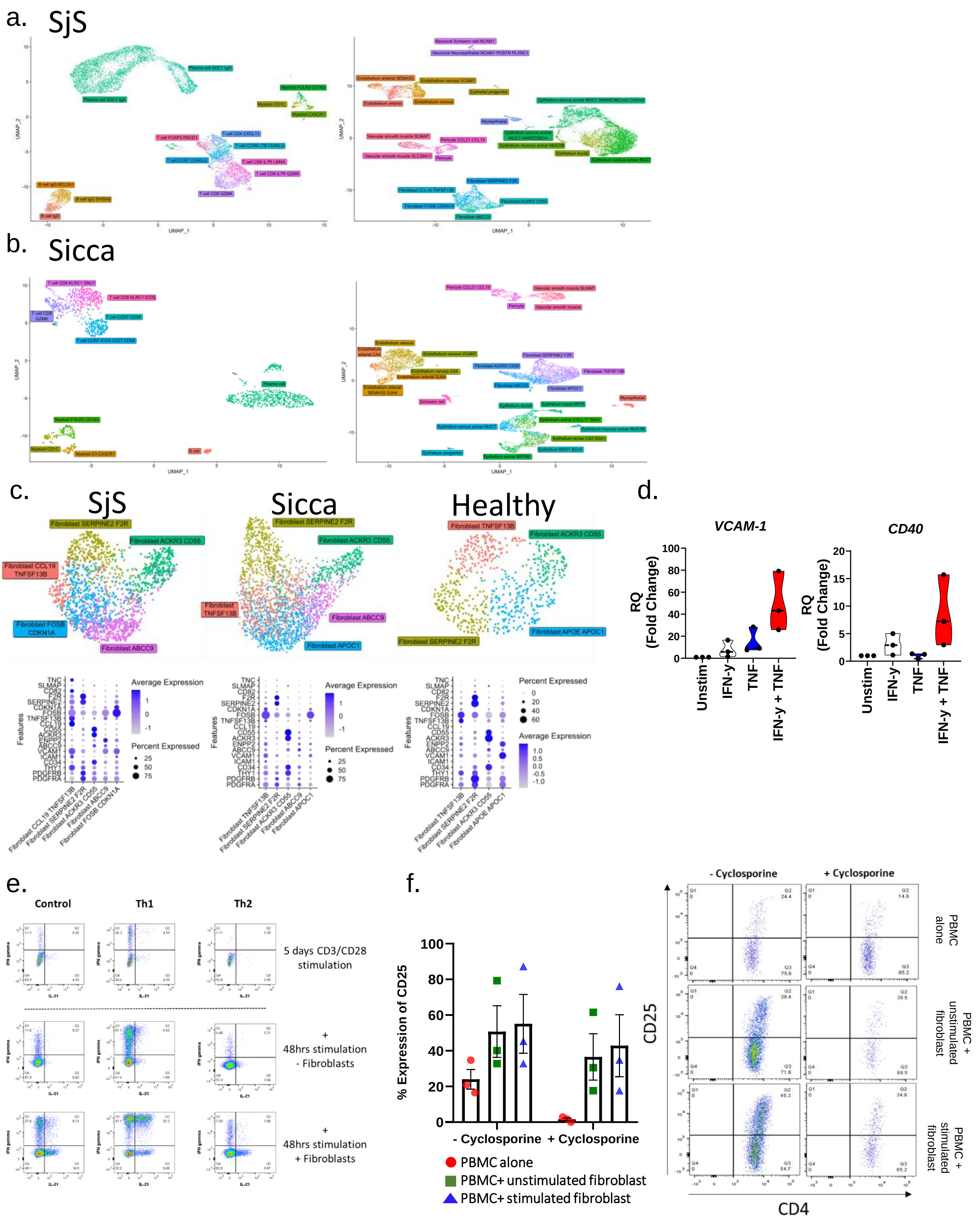

Supplementary figure 4. a and b, UMAP reduction of SjS and sicca 10x data annotated with all cluster identities. **c,** Analysis of minor salivary gland fibroblast populations in disease and health discovers similar clusters across states. UMAPs highlight discovered clusters in each condition. Dotplots display the expression of marker genes for subsets at different conditions. **d,** Gene expression from *in vitro* cytokine stimulated salivary gland fibroblasts (n=3) to acquire features of immunofibroblasts or CCL19 TNFSF13B fibroblast cluster. The cytokine treatment is indicated in the graph. RQ= Relative quantification. **e,** Human naïve CD4+ T cells were activated for 5 days using ImmunoCultTM Human CD3/CD28 T Cell Activator (StemCellTM Technologies) in the presence of 100U/mL IL-2. T cell differentiation was induced using ImmunoCultTM human Th1/Th2 differentiation supplements (StemCellTM Technologies). Cells were then washed and incubated in the presence of either unstimulated or TNFα/IL-13/ LTα1β2 stimulated SjS fibroblasts for 48 hours. TNFα/IL-13/ LTα1β2 cytokine milieu to induce TLS fibroblasts previously described in[5] . For the detection of cytokine expression, T cells were re-stimulated for 4 hours with 50ng/mL PMA, 1μM Ionomycin in the presence of 10μg/mL brefeldin A. Cells were then fixed/permeabilised and stained for indicated cytokines. Representative flow cytometry data of 3 independent experiments. f, FACS analysis of CD4+ CD25+ expression in healthy human donor PBMC populations in the absence of presence of unstimulated or stimulated SjS fibroblasts in vitro after 48hrs. **f.** Representative plots of CD25 expression on human CD4+ T cells in the presence or absence of fibroblasts whilst treated with Cyclosporine (15 ug/ml).

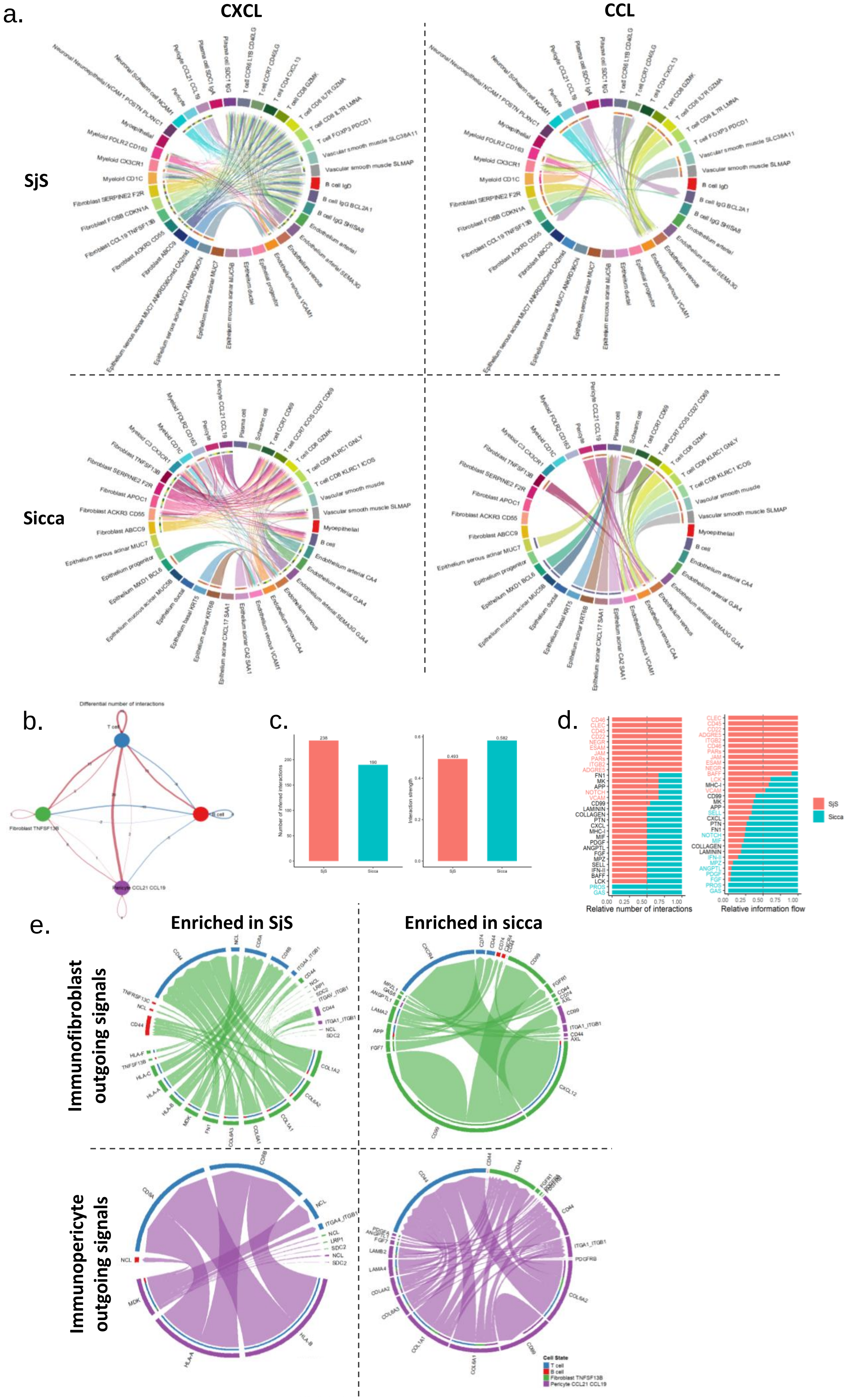

Supplementary figure 5. a, Cellchat analysis chord diagrams showing sender and receiver populations for ligands included in CXCL or CCL pathways in both SjS and Sicca. Inner bars illustrate receiving populations and the proportion of the bar illustrates signal strength. **b, c, d, and e** show comparative cellchat analysis of immunofibroblasts (fibroblast TNFSF13B), pericyte CCL21 CCL19, T cell, and B cell populations. T cell and B cell populations contained all subclusters of T cells and B cells in the granular analysis. **b,** circle plot of differential number of interactions in SjS versus Sicca. Red edges indicate increased number of interactions in SjS and blue in Sicca. Annotation provides the number of differential interactions. **c,** barplots providing the total number and strength of interactions in SjS versus Sicca. **d,** barplots of the relative number of interactions and of relative information flow in SjS versus Sicca by signalling pathways. Highlighted names indicate pathways with statistically significant differences. **e,** chord plots highlighting enriched outgoing signals from immunofibroblasts or CCL21 CCL19 pericytes in SjS and sicca.

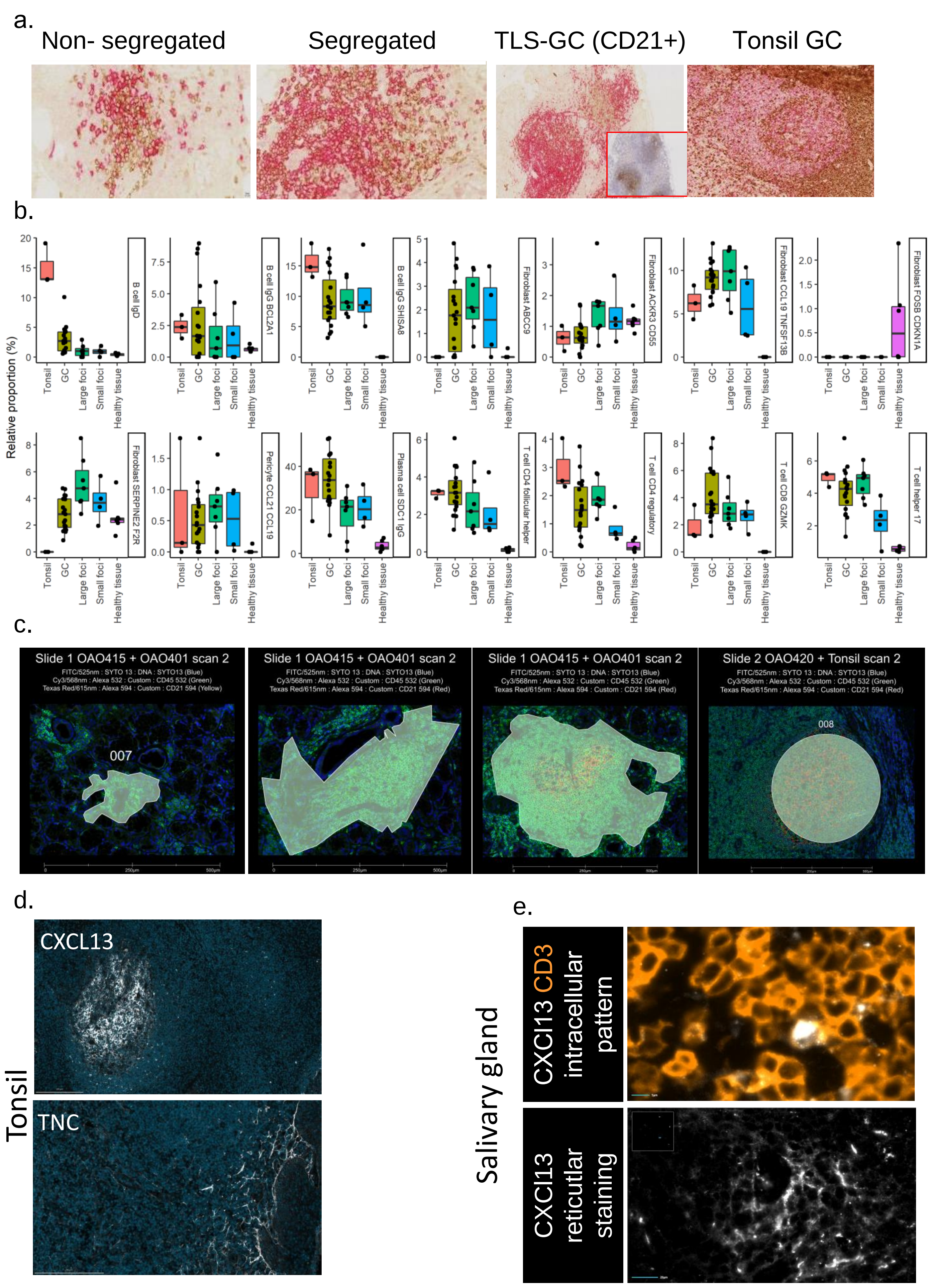

Supplementary figure 6: a, Representative images of immunohistochemical staining of stages of aggregate maturation with CD3 stained brown and CD20 stained red. **b**, Extended plotting of CibersortX imputation of cell state proportions in bulk RNA-sequencing data derived from microdissection of SjS salivary gland tissue. Boxplots show the median, first and third quartiles, and whiskers extend to the largest value no further than 1.5 times the interquartile range. Tonsil = 3, tertiary lymphoid structure germinal center (TLS GC) = 19, Segregated = 7, Non segregated = 4, healthy control = 5 **c,** Representative marked up images showing ROI selection for the *GeoMx® DSP* protein assay. **d,** Single-colour controls for immunofluorescence staining of CXCL13 and TNC in tonsil, Scale bar=200µm. **e,** High magnification imaging of salivary gland detailing CXCL13 staining patterns in reticular and intracellular patterns, Scale bar=200µm.

H&E images of salivary gland biopsies from patients used for scRNAseq analysis

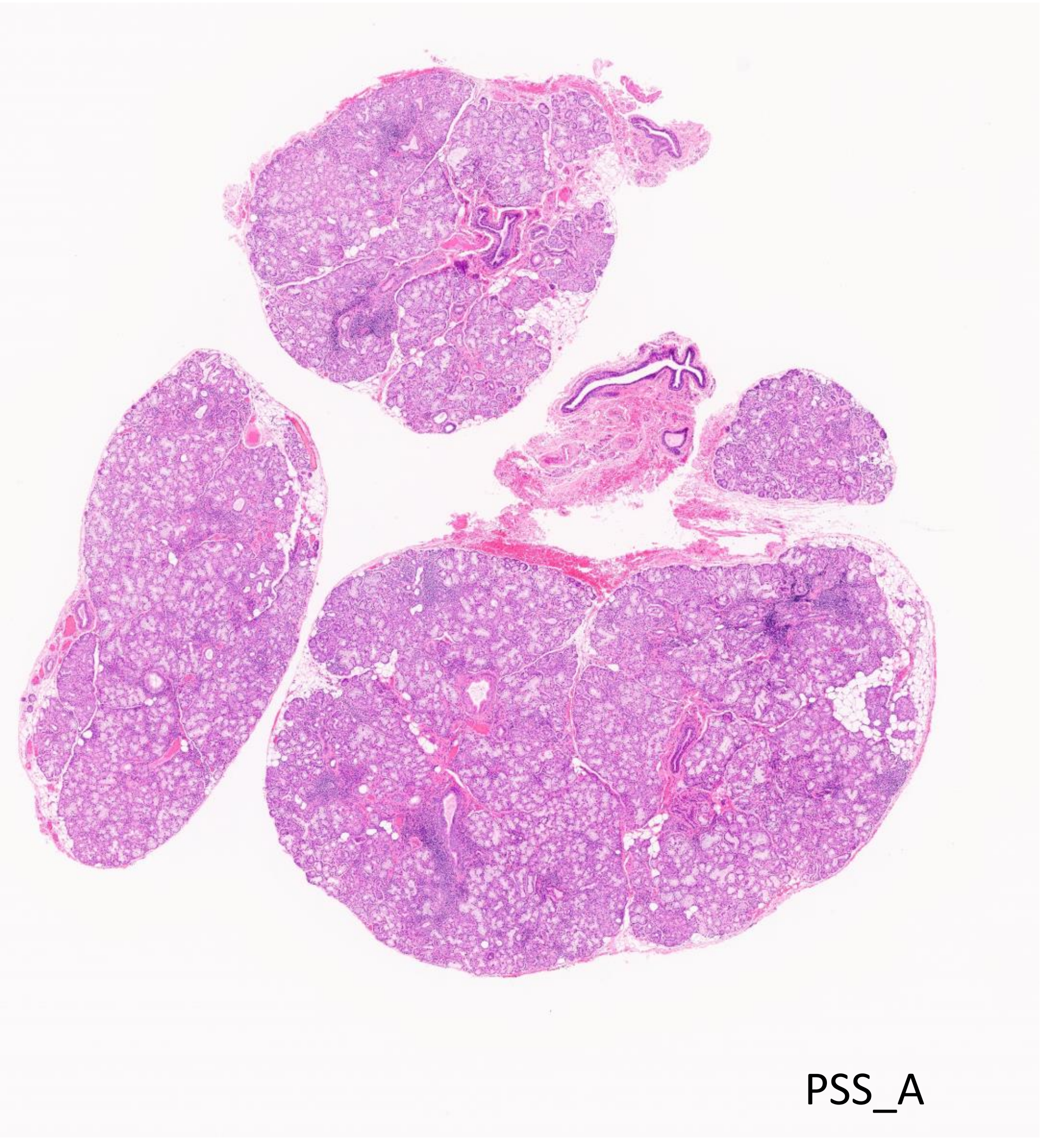

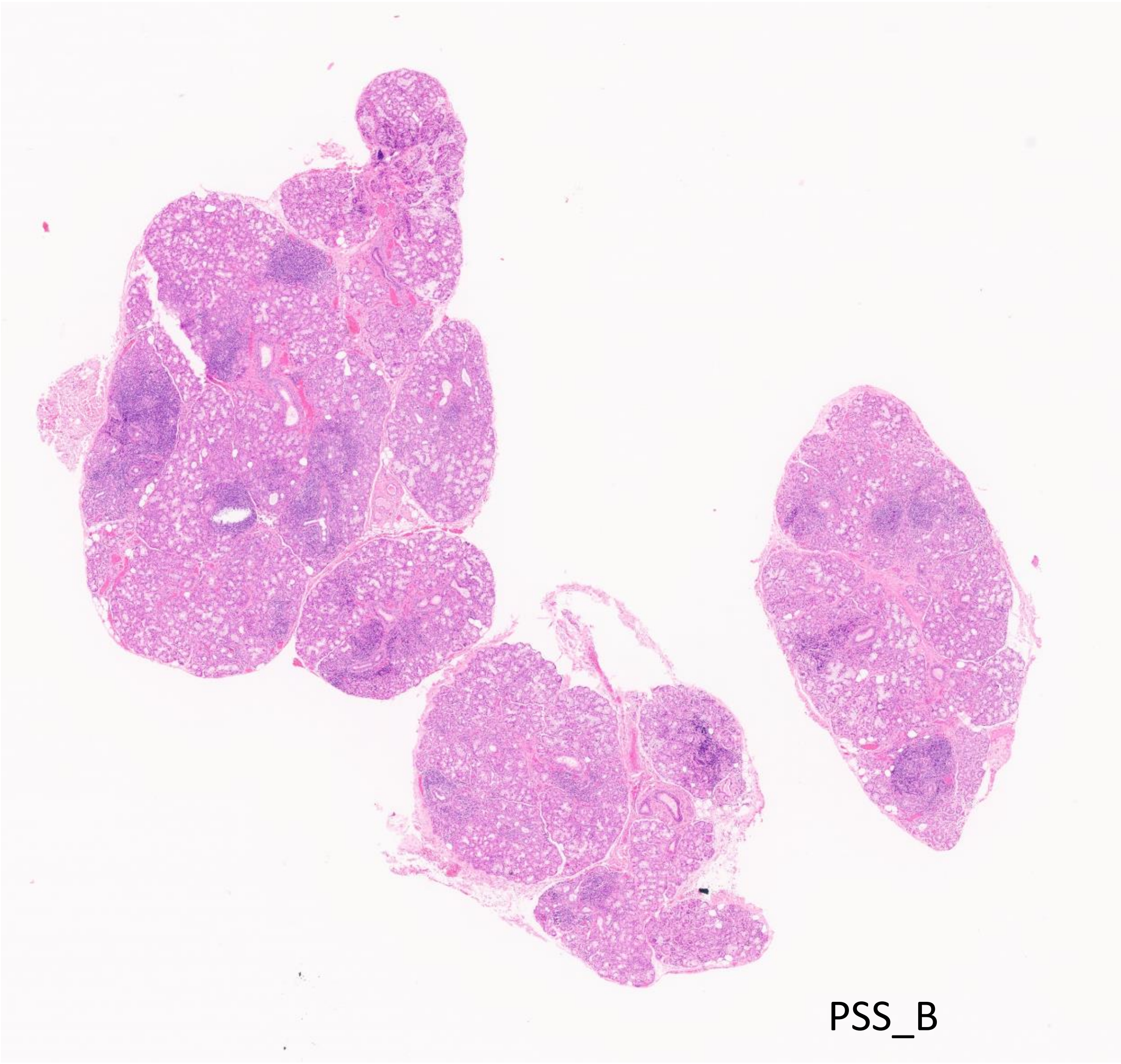

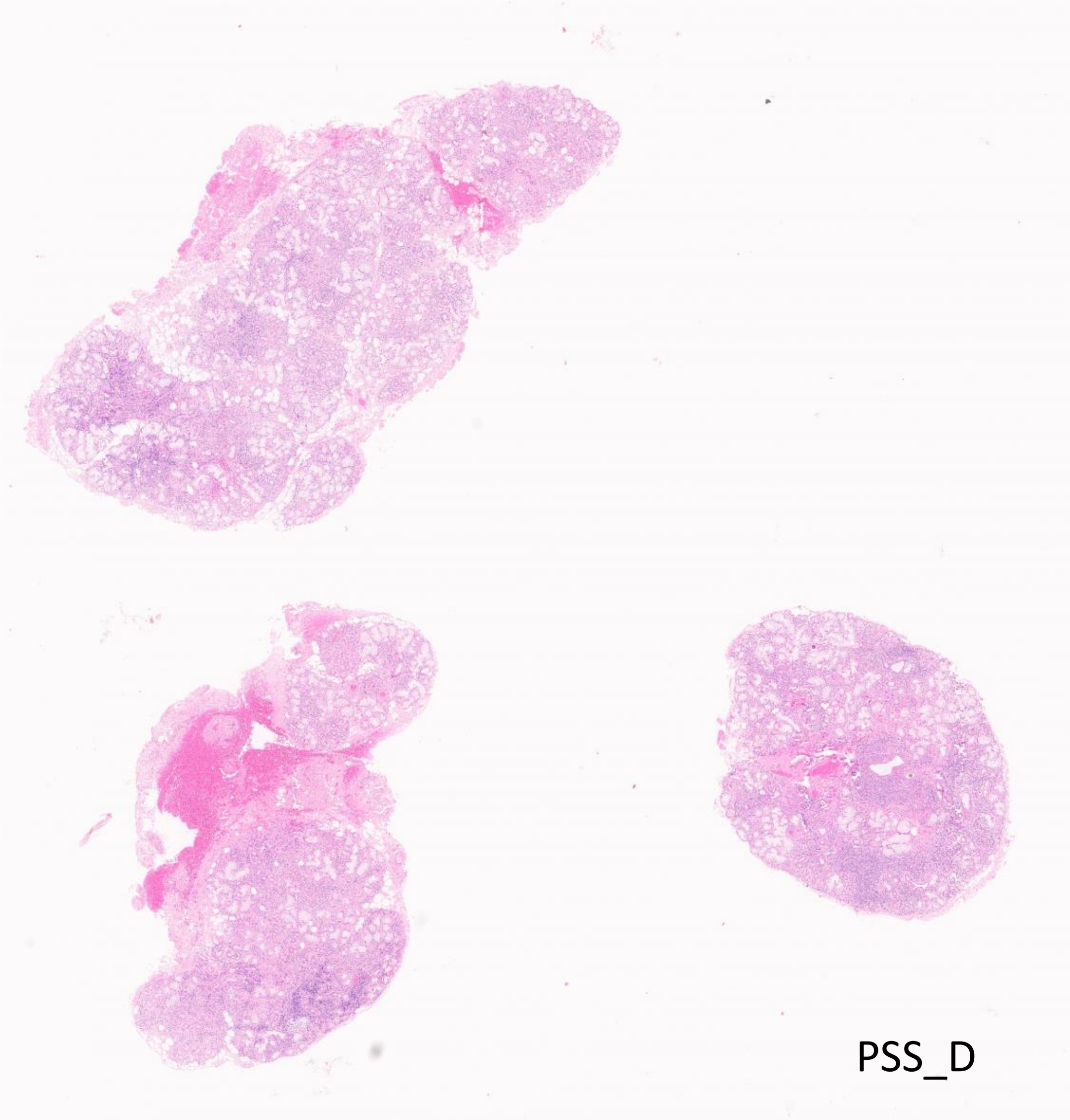

PSS_D

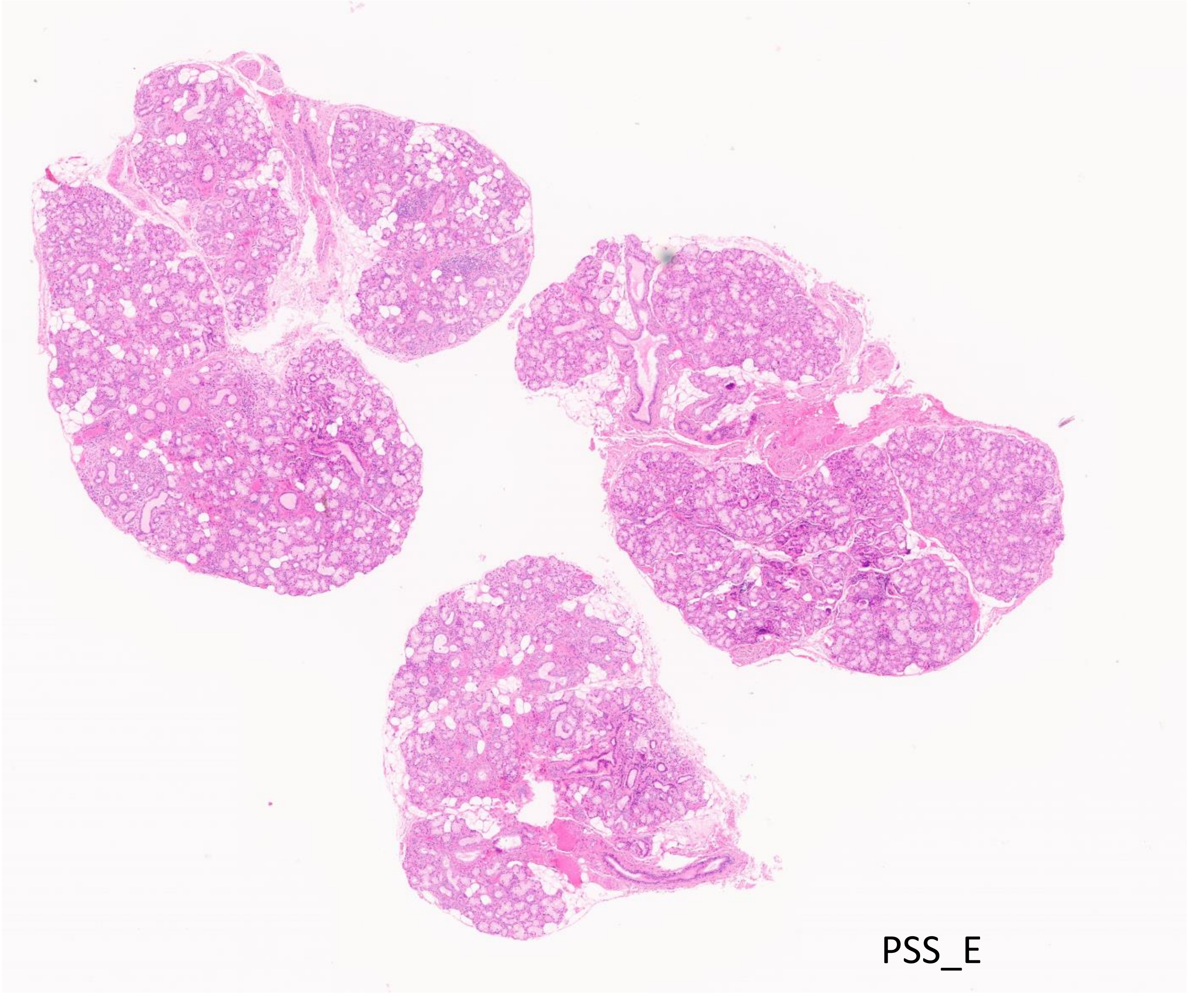

PSS_E

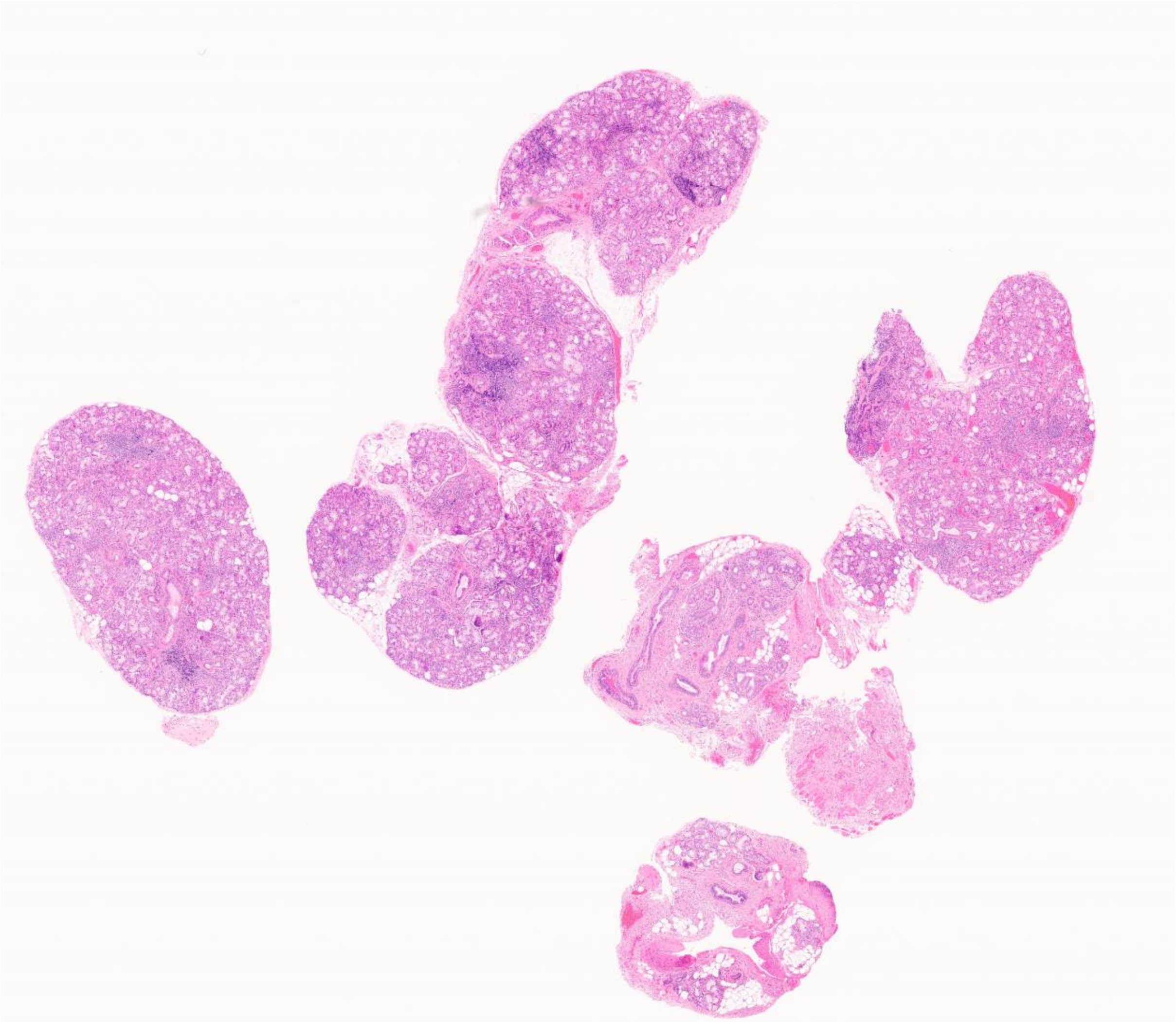

PSS_F

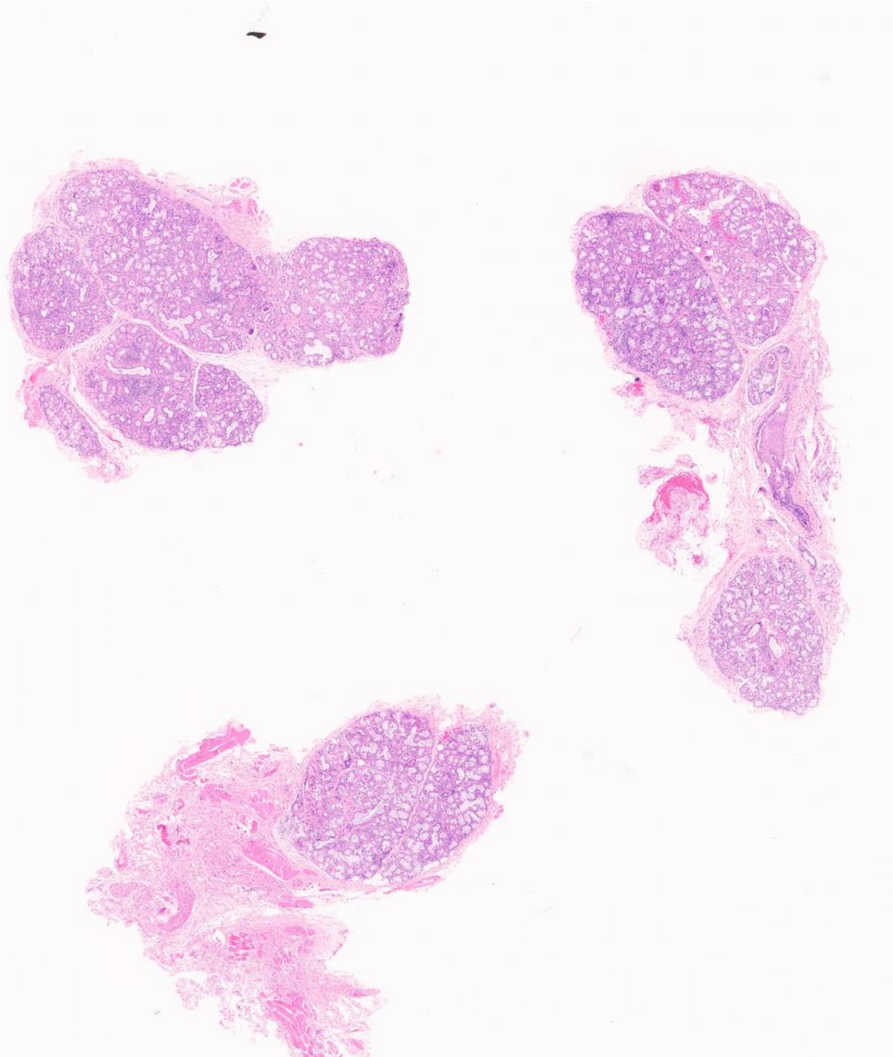

PSS_G

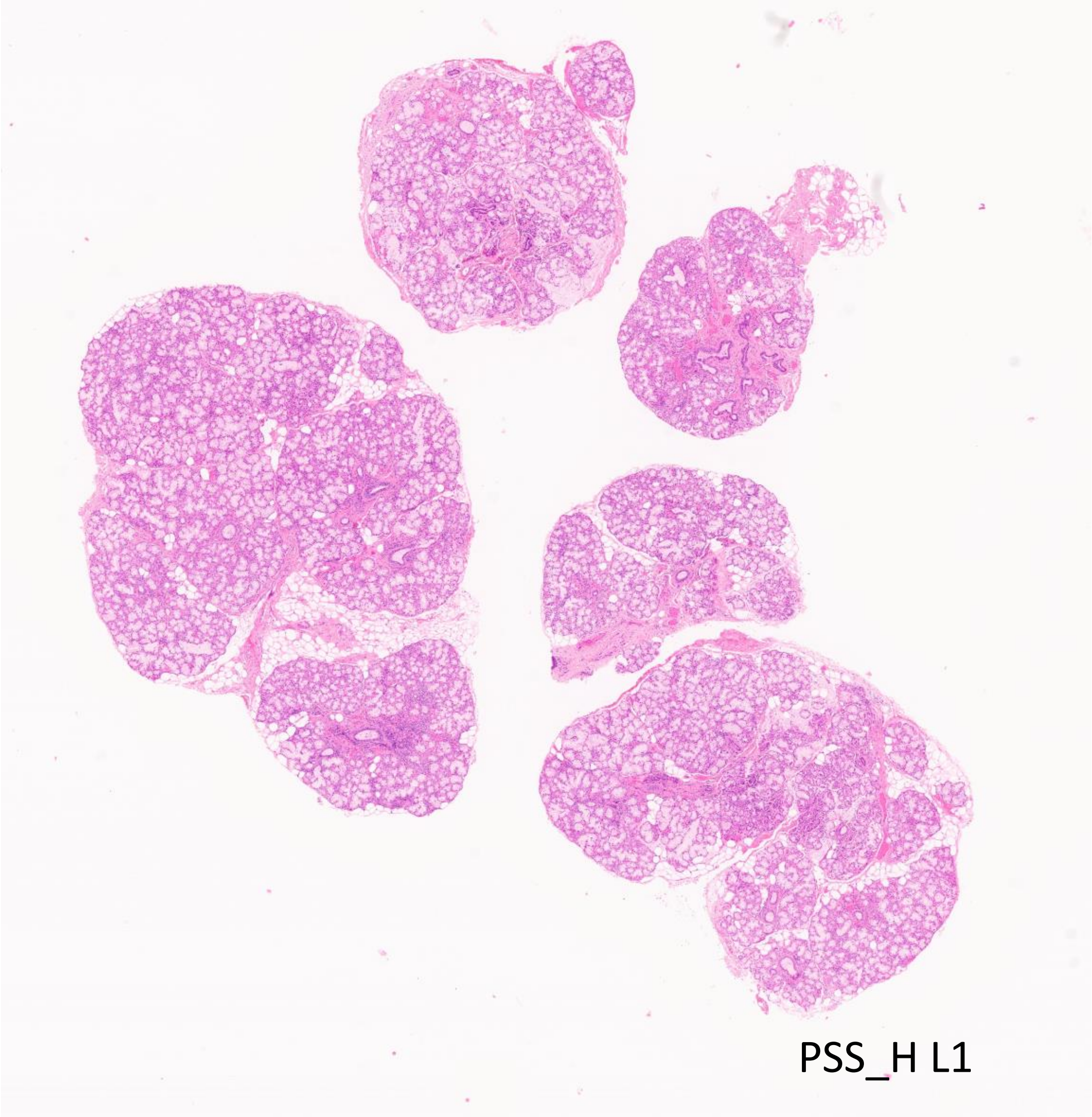
PSS_H L1

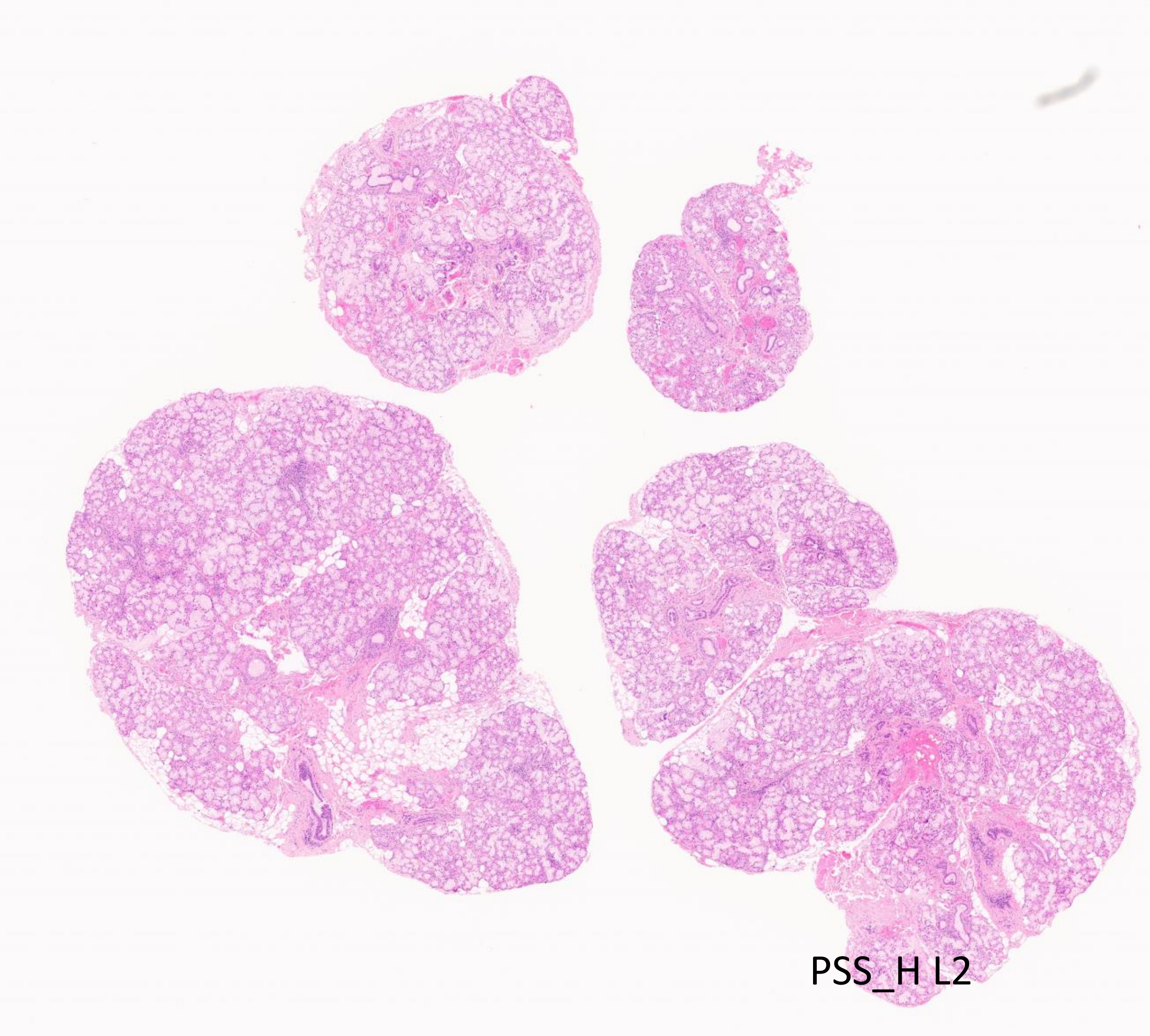
PSS_H L2

H&E images of salivary gland biopsies from SjS patients used for scRNAseq analysis

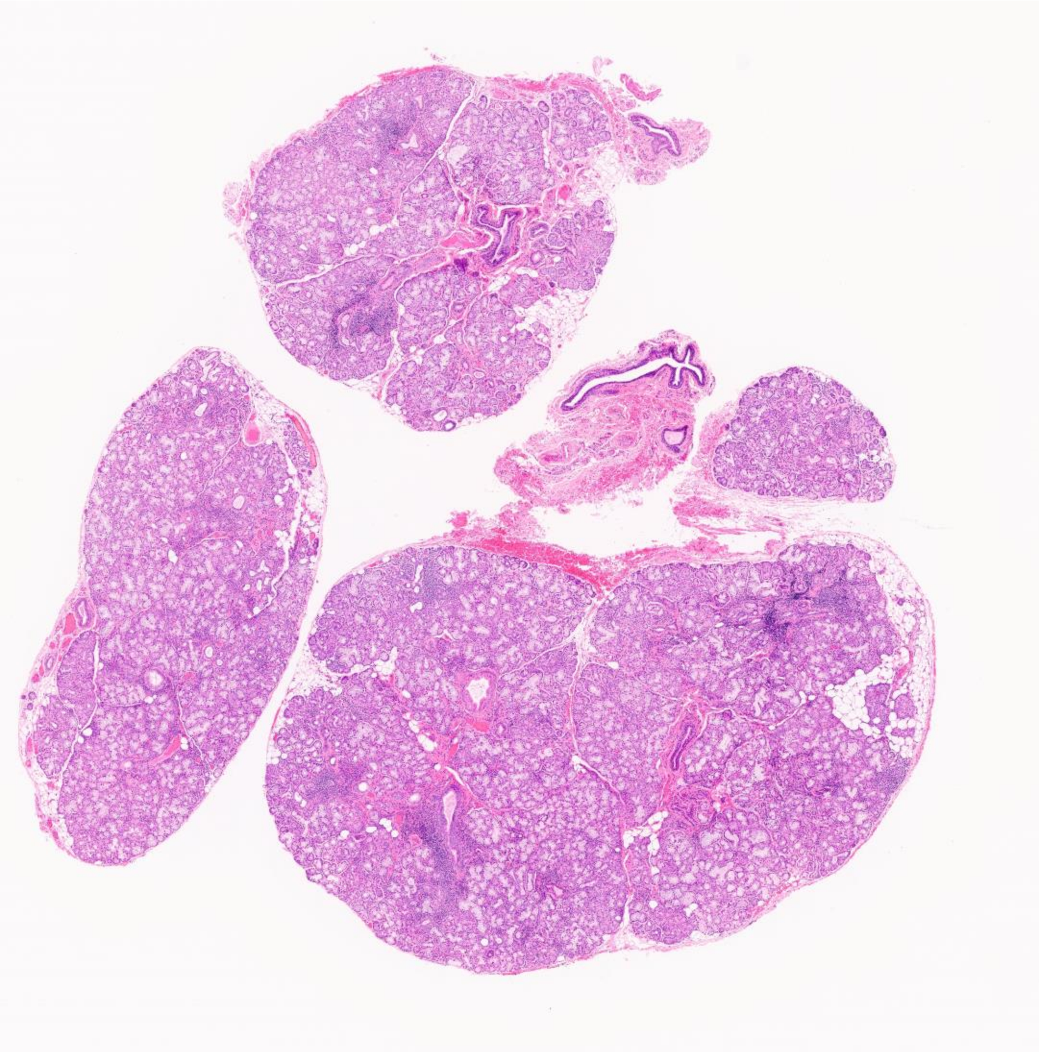

PSS_A

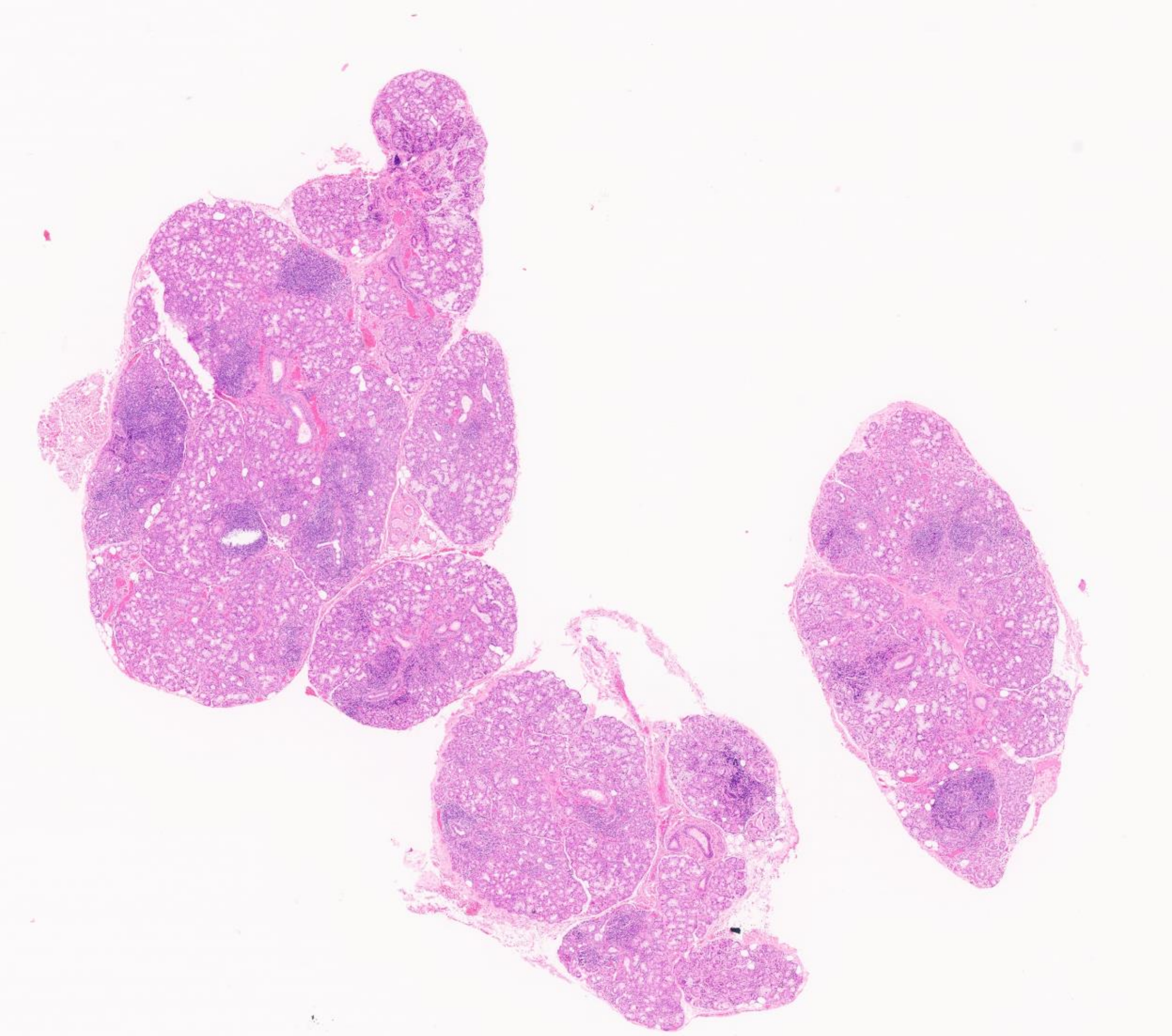

PSS_B

H&E images of salivary gland biopsies from Sicca patients used for scRNAseq analysis

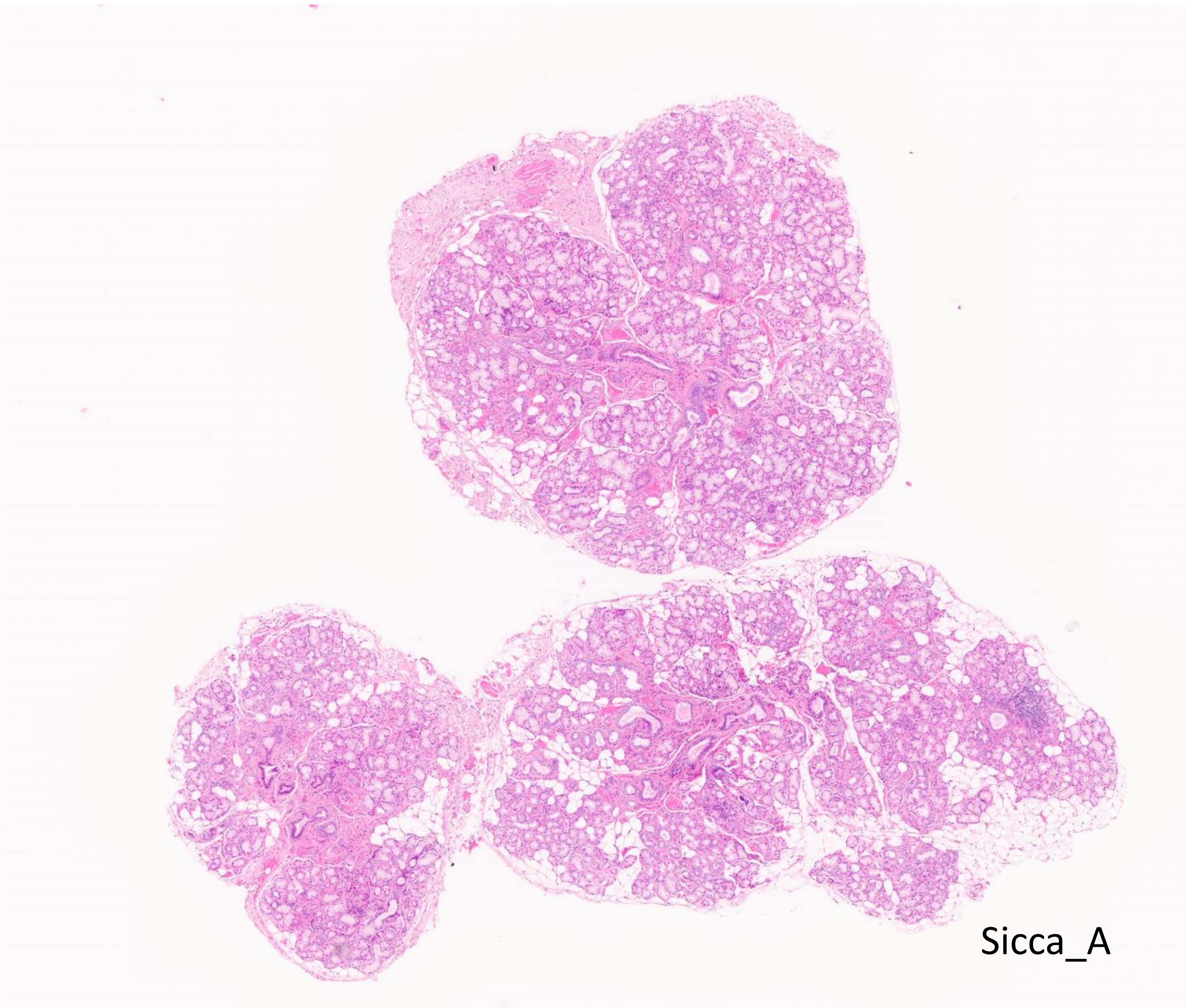

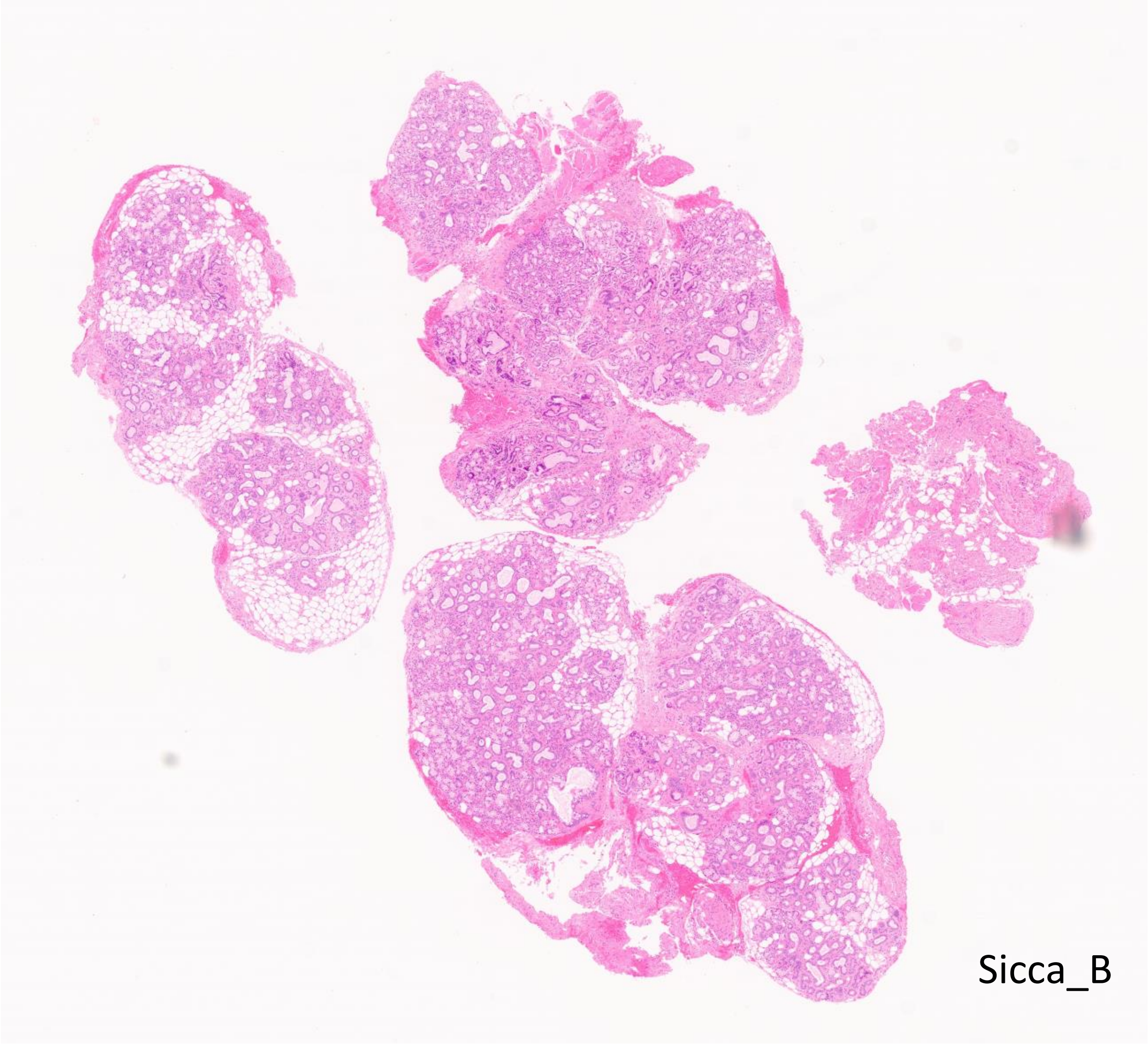

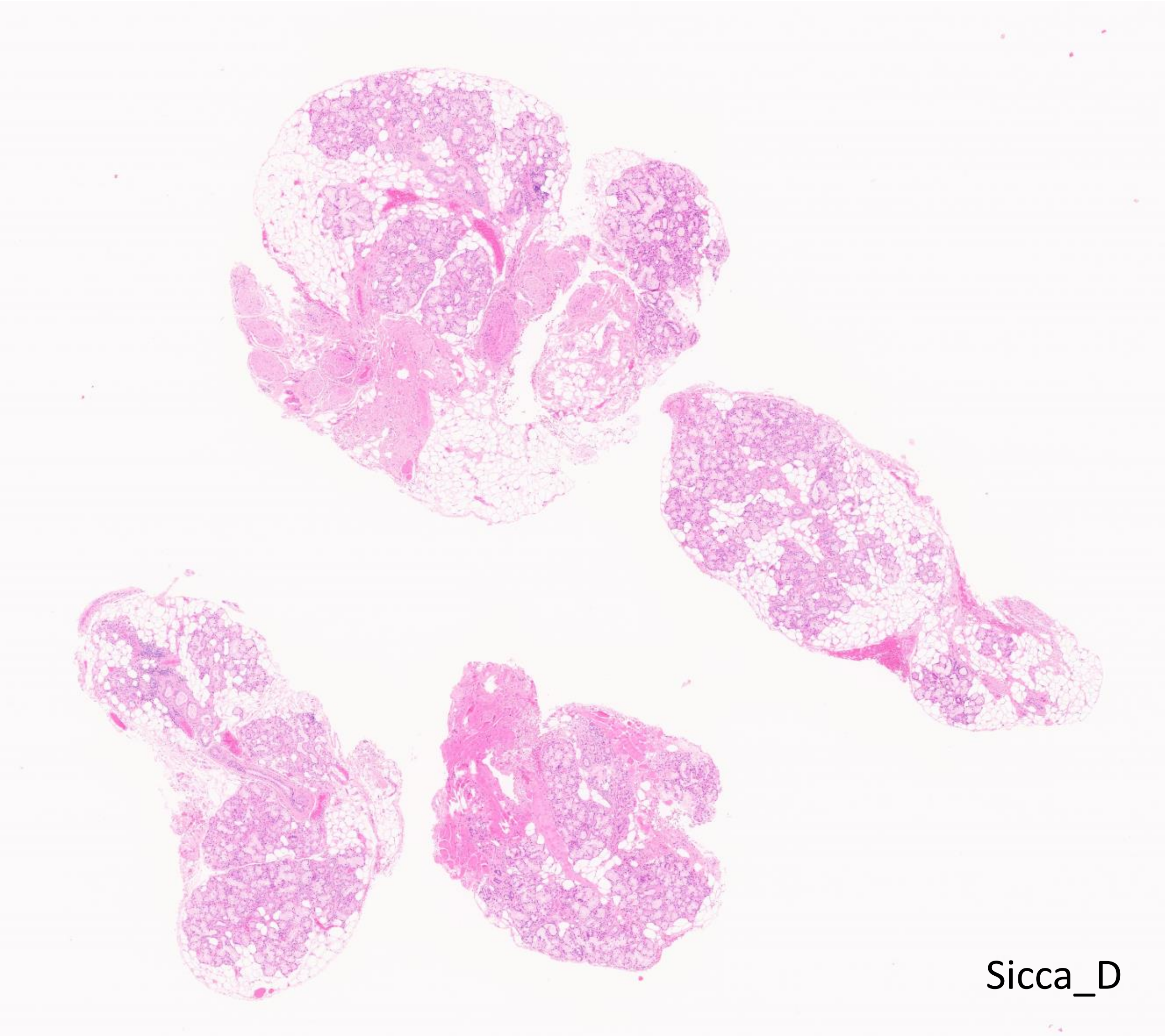
Sicca_D

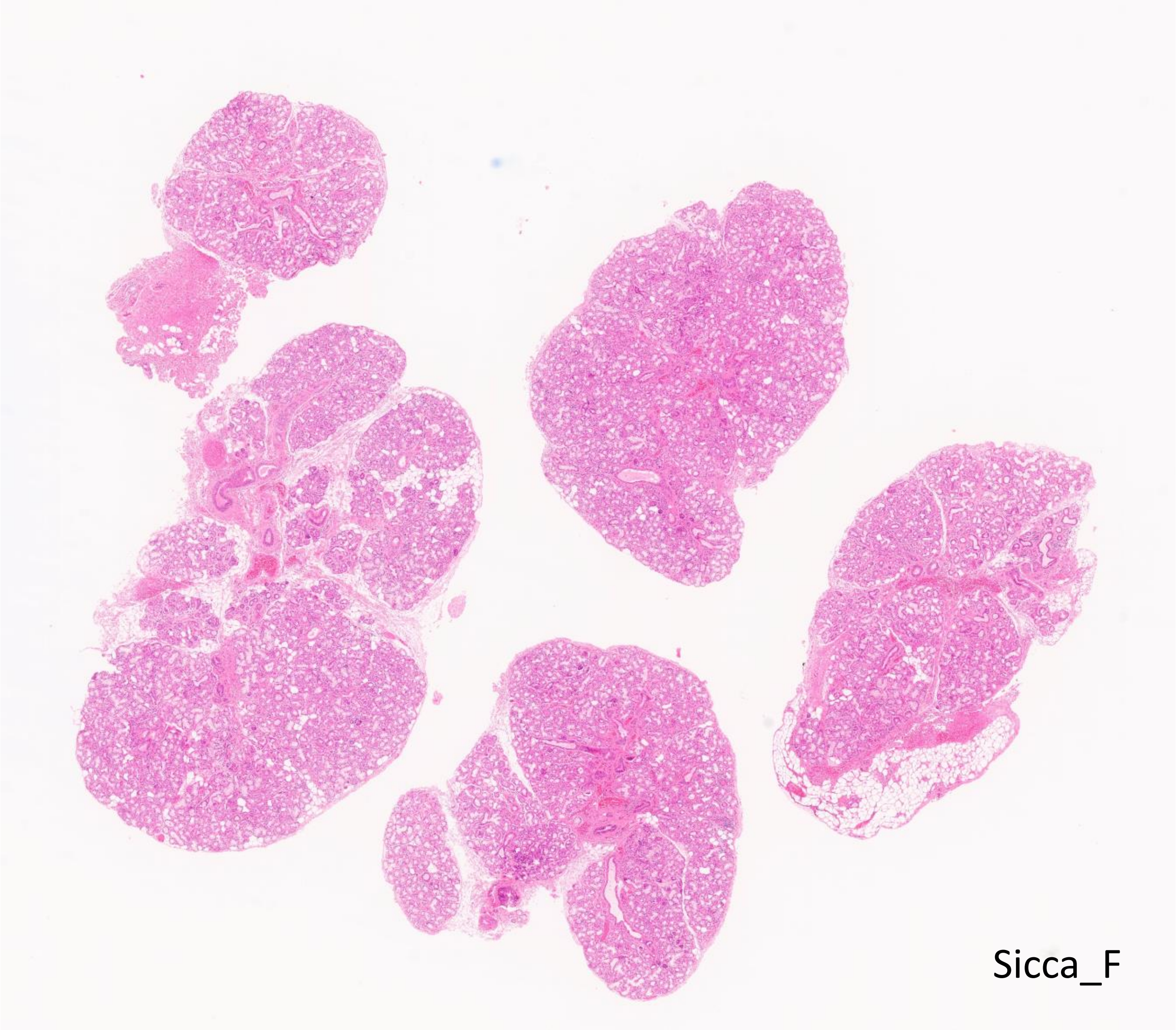
Sicca_F

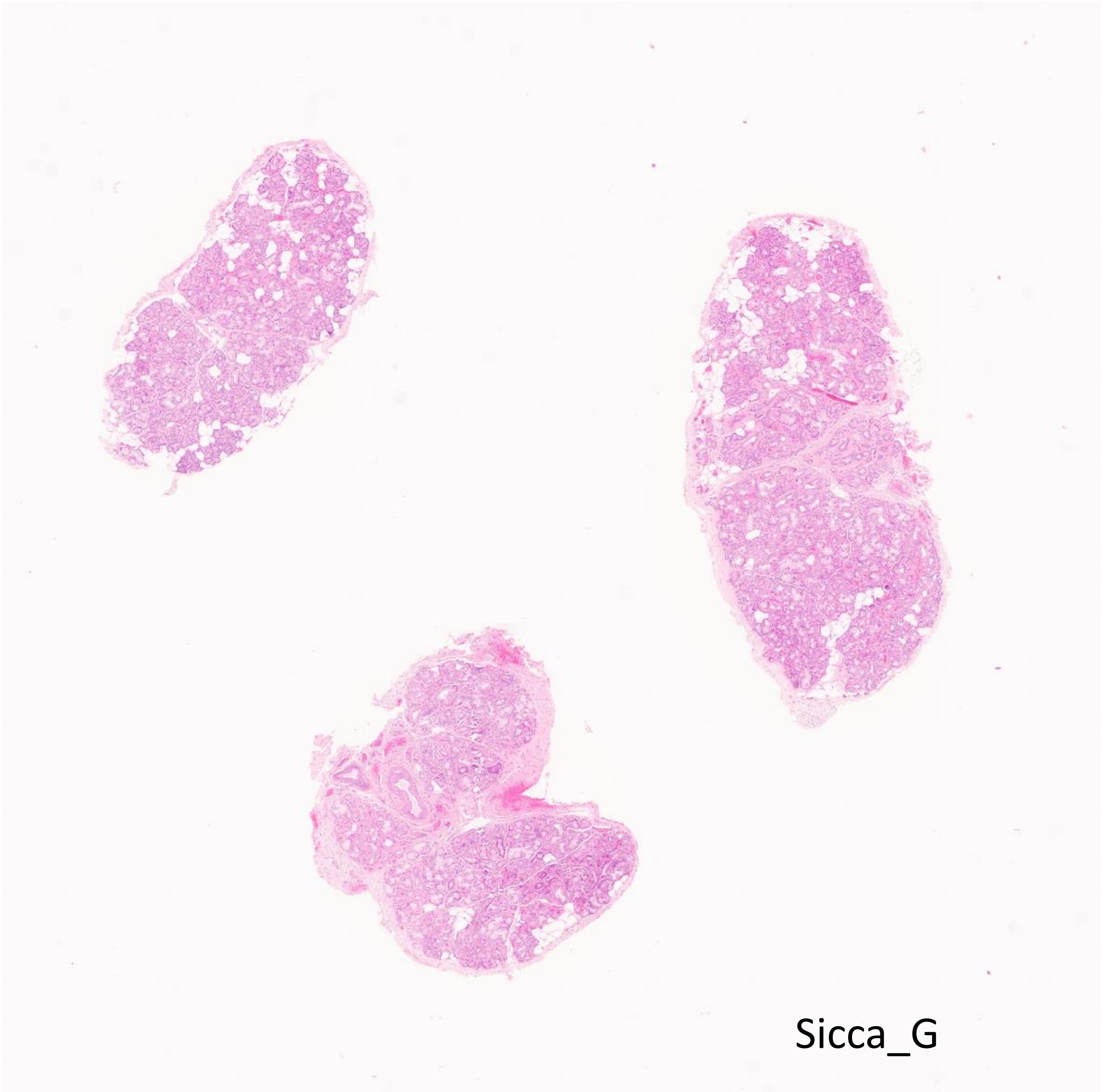
Sicca_G

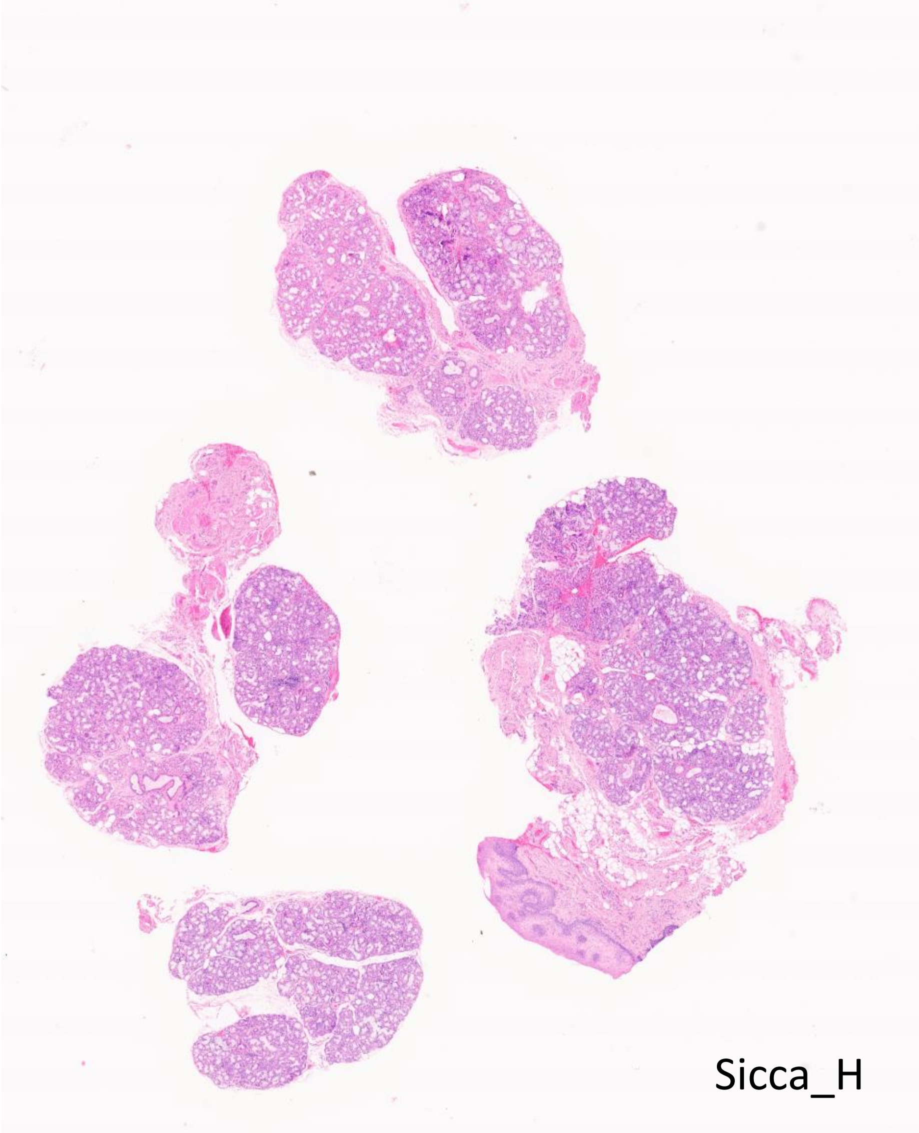
Sicca_H
