# Supplementary material for: Molecular and spatial analysis of tertiary lymphoid structures in Sjogren’s syndrome
Source: Nat Commun. 2025 Jan 2;16:5. doi: 10.1038/s41467-024-54686-0 (PMC11697438; doi:10.1038/s41467-024-54686-0)
Supplement: Supplementary file 2 — Description of Additional Supplementary Files [file 41467_2024_54686_MOESM2_ESM.pdf]

Description of Additional Supplementary Files

File Name: Supplementary Data 1
Description: Overview of patient data used for single cell analysis.

File Name: Supplementary Data 2
Description: List of differentially expressed genes for global clusters.

File Name: Supplementary Data 3
Description: List of differentially expressed genes for fibroblast and mural cell clusters.
