# Supplementary material for: Molecular and spatial analysis of tertiary lymphoid structures in Sjogren’s syndrome
Source: Nat Commun. 2025 Jan 2;16:5. doi: 10.1038/s41467-024-54686-0 (PMC11697438; doi:10.1038/s41467-024-54686-0)
Supplement: Supplementary file 6 — Reporting Summary [file 41467_2024_54686_MOESM6_ESM.pdf]

nature portfolio

Corresponding author(s): Francesca Barone

Last updated by author(s): 24/09/24

Reporting Summary

Nature Portfolio wishes to improve the reproducibility of the work that we publish. This form provides structure for consistency and transparency in reporting. For further information on Nature Portfolio policies, see our Editorial Policies and the Editorial Policy Checklist.

Statistics

For all statistical analyses, confirm that the following items are present in the figure legend, table legend, main text, or Methods section.

n/a	Confirmed	
☐	☒	The exact sample size (*n*) for each experimental group/condition, given as a discrete number and unit of measurement
☒	☐	A statement on whether measurements were taken from distinct samples or whether the same sample was measured repeatedly
☐	☒	The statistical test(s) used AND whether they are one- or two-sided<br>*Only common tests should be described solely by name; describe more complex techniques in the Methods section.*
☐	☒	A description of all covariates tested
☐	☒	A description of any assumptions or corrections, such as tests of normality and adjustment for multiple comparisons
☐	☒	A full description of the statistical parameters including central tendency (e.g. means) or other basic estimates (e.g. regression coefficient) AND variation (e.g. standard deviation) or associated estimates of uncertainty (e.g. confidence intervals)
☐	☒	For null hypothesis testing, the test statistic (e.g. *F*, *t*, *r*) with confidence intervals, effect sizes, degrees of freedom and *P* value noted<br>*Give P values as exact values whenever suitable.*
☒	☐	For Bayesian analysis, information on the choice of priors and Markov chain Monte Carlo settings
☒	☐	For hierarchical and complex designs, identification of the appropriate level for tests and full reporting of outcomes
☒	☐	Estimates of effect sizes (e.g. Cohen's *d*, Pearson's *r*), indicating how they were calculated

Our web collection on statistics for biologists contains articles on many of the points above.

Software and code

Policy information about availability of computer code

Data collection: Detailed in methods

Data analysis: Reads for each sample were aligned to the hg38 transcriptome and quantified using Cellranger v3.0.2 on the University of Birmingham BlueBEAR High Performance Computing service. Potential doublets were identified and removed using Scrublet v0.2.1 prior to sample aggregation and UMI normalisation using scripts adapted from the Sansom lab repository (https://github.com/sansomlab). scRNAseq analysis completed using R v3.6.1 and Seurat v.2.3.4. Harmony v1.0 used to integrate data. Cluster stability was assessed using Clustree v0.4.3. Final clustering was completed in R v4.0.3 and Seurat v4.0.2. Slingshot v1.8 used to run trajectory analysis. Gene-set overrepresentation analysis run using gsfisher v0.2.CellChat v1.4 used to run ligand-receptor. Pseudobulk analysis completed using DESeq2 v1.30.1.

Bulk seq read adapters were trimmed with trimmomatic v0.39, reads aligned to the hg38 transcriptome with STAR v2.7.2b, and counts summed using featureCounts in Subread v2.0.1 using the University of Birmingham BlueBEAR High Performance Computing service. R v4.0.3 and DESeq2 v1.30.1 were used for downstream analysis. Data variance stabilising transformed data using limma v3.46.0

Deconvolution of sequencing data completed using the web interface to CibersortX. CellChat v1.4 was used to run ligand-receptor analysis on the single-cell sequencing dataset. SCENIC v1.3.1 was used with default parameters to infer regulon activity within the single-cell RNA sequencing data.

Multispectral images were acquired at using the Vectra Polaris Automated Quantitative Pathology Imaging System (Akoya) or COMET (Lunaphore). MoTIF settings were used for multispectral image acquisition. Multispectral image processing of multiplex IHC stains was performed using Phenochart (version 1.0.11/Akoya) and inForm Image Analysis Software (version 2.3, Akoya) or Lunaphore image viewer.

COMET analysis completed using Lunaphore COMET Explorer software and Lunaphore COMET Viewer v2. Segmentation completed with CellSeg. Cell phenotyping was performed using CELESTA and MetaCyto.

Microdissection was completed using the PALM Robo Software V.4.6 software

GEOMX analysis completed using Geomx software, R v4.0.3 and the pheatmap v1.0.12 package.

For manuscripts utilizing custom algorithms or software that are central to the research but not yet described in published literature, software must be made available to editors and reviewers. We strongly encourage code deposition in a community repository (e.g. GitHub). See the Nature Portfolio guidelines for submitting code & software for further information.

Data

Policy information about availability of data

All manuscripts must include a data availability statement. This statement should provide the following information, where applicable:

- Accession codes, unique identifiers, or web links for publicly available datasets
- A description of any restrictions on data availability
- For clinical datasets or third party data, please ensure that the statement adheres to our policy

The Single-cell RNA sequencing data generated in this study have been deposited in the Gene Expression Omnibus database under accession code GSE272409 [https://www.ncbi.nlm.nih.gov/geo/query/acc.cgi?acc=GSE272409]. The Bulk RNA sequencing data generated in this study have been deposited in the Gene Expression Omnibus database under accession code GSE272410 [https://www.ncbi.nlm.nih.gov/geo/query/acc.cgi?acc=GSE272410].

Research involving human participants, their data, or biological material

Policy information about studies with human participants or human data. See also policy information about sex, gender (identity/presentation), and sexual orientation and race, ethnicity and racism.

Reporting on sex and gender	Samples are derived from both male and female (biological sex) patients. However, the data are bias towards samples from the female sex reflecting the bias of Sjogren's disease in the general population. Additionally, the sex distribution of samples is restricted to the availability of samples within the cohort. Sample metadata are available in the supplementary data.
Reporting on race, ethnicity, or other socially relevant groupings	n/a
Population characteristics	Patient data is available in TableS1
Recruitment	Labial minor salivary gland samples were obtained from patients recruited in the Optimising Assessment in Sjögren's Syndrome (OASIS) cohort which recruits new patients attending the multidisciplinary Sjögren's clinic at the Queen Elizabeth Hospital Birmingham, UK for assessment. Sjögren's syndrome patients had a physician diagnosis of primary Sjögren's syndrome and fulfilled the 2016 ACR/EULAR classification criteria. Participants with non-Sjögren's sicca syndrome had signs and/or symptoms of dryness but did not have a physician diagnosis of SS or fulfill 2016 classification criteria. Salivary gland biopsy samples were divided in two: one for the scRNAseq study and the second for histological analysis to confirm diagnosis. Histological diagnosis is reported as presence of focal lymphocytic sialadenitis (FLS, suggestive of Primary Sjögren's Syndrome, PSS) or non-specific chronic sialadenitis (NSCS), in the case of non-Sjögren's sicca syndrome.
Ethics oversight	All OASIS participants provided written informed consent and the study was approved by the Wales Research Ethics Committee 7 (WREC 7) formerly Dyfed Powys REC; 13/WA/0392.

Note that full information on the approval of the study protocol must also be provided in the manuscript.

Field-specific reporting

Please select the one below that is the best fit for your research. If you are not sure, read the appropriate sections before making your selection.

[x] Life sciences [ ] Behavioural & social sciences [ ] Ecological, evolutionary & environmental sciences

For a reference copy of the document with all sections, see nature.com/documents/nr-reporting-summary-flat.pdf

Life sciences study design

All studies must disclose on these points even when the disclosure is negative.

Sample size	scRNAseq; n=7 Sjogren's disease samples, n=6 Sicca syndrome samples.<br>Murine LTBr KO; wt=4, ltbr-/-=4.<br>Microdissection sequencing; Non-segregated=4, Segregated=7, TLS-GC=18, Tonsil-GC=3.<br>GEOMX proteomics; Non-segregated=10, Segregated=22, TLS-GC=20, Tonsil-GC=6.<br>No statistics was used to predetermine the sample size, the numbers used in the study are based on previous work done in the group.

Data exclusions	scRNAseq; 1 Sjogren's disease and 2 Sicca syndrome samples excluded due to low quality as assessed by the number of unique molecular identifiers, number of genes detected, excessive mitochondrial gene percentage, or paucity of cells. Only background noise was supplied as a covariate to the modelling of differential expression of the single-cell RNA sequencing data
Replication	Experimental and data replicates mentioned in each figure legend.
Randomization	Randomization not required.
Blinding	Not required due to hypothesis discovery and nature of the study.

Reporting for specific materials, systems and methods

We require information from authors about some types of materials, experimental systems and methods used in many studies. Here, indicate whether each material, system or method listed is relevant to your study. If you are not sure if a list item applies to your research, read the appropriate section before selecting a response.

Materials & experimental systems

n/a	Involved in the study	
☐	☒	Antibodies
☒	☐	Eukaryotic cell lines
☒	☐	Palaeontology and archaeology
☐	☒	Animals and other organisms
☒	☐	Clinical data
☒	☐	Dual use research of concern
☒	☐	Plants

Methods

n/a	Involved in the study	
☒	☐	ChIP-seq
☐	☒	Flow cytometry
☒	☐	MRI-based neuroimaging

Antibodies

Antibodies used	Details of all the antibodies used in this manuscript are in the methods section.
Validation	Antibodies used in this manuscript are well established clones used across different studies and extensively validated by vendors. Where necessary antibody validation has been provided in the in the supplementary figures.

Animals and other research organisms

Policy information about studies involving animals; ARRIVE guidelines recommended for reporting animal research, and Sex and Gender in Research

Laboratory animals	C57BL/6 wild-type (wt) female mice were purchased from Charles River. Ltbr-/- female mice were provided by Jorge Caamano. All mice were maintained under specific pathogen-free conditions in the Biomedical Service Unit at the University of Birmingham according to Home Office and local ethics committee regulations.
Wild animals	n/a
Reporting on sex	Female mice were used in the study to reflect the strong female bias in the human disease (Sjogren Syndrome).
Field-collected samples	n/a
Ethics oversight	All mice were experiments were conducted in the Biomedical Service Unit at the University of Birmingham according to Home Office and local ethics committee regulations (University of Birmingham), under license no. P4B291FAA.

Note that full information on the approval of the study protocol must also be provided in the manuscript.

Flow Cytometry

Plots

Confirm that:

- [x] The axis labels state the marker and fluorochrome used (e.g. CD4-FITC).
- [x] The axis scales are clearly visible. Include numbers along axes only for bottom left plot of group (a 'group' is an analysis of identical markers).
- [x] All plots are contour plots with outliers or pseudocolor plots.
- [x] A numerical value for number of cells or percentage (with statistics) is provided.

Methodology

Sample preparation	Detailed description in methods section
Instrument	Information provided in methods section
Software	FlowJo was used to analyze flow cytometry data
Cell population abundance	Proportion of each population has been highlighted in the flow cytometry plots
Gating strategy	Gating strategy has been provided in the supplementary figures

- [x] Tick this box to confirm that a figure exemplifying the gating strategy is provided in the Supplementary Information.
